# Regression analysis of interval-censored competing risks data with missing causes of failure: A direct likelihood approach

**DOI:** 10.1177/09622802261420820

**Published:** 2026-03-04

**Authors:** Yichen Lou, Yuqing Ma, Liming Xiang, Jianguo Sun

**Affiliations:** 1 School of Physical and Mathematical Sciences, 534931 Nanyang Technological University, Singapore; 2 12510 Jiangsu Hengrui Pharmaceuticals, Co., Ltd., Shanghai, China; 3 Department of Statistics, 2628 University of Missouri, Columbia, MO, USA

**Keywords:** Competing risks, cumulative incidence function, interval-censoring, mixture models, sieve maximum likelihood estimation

## Abstract

Regression analysis of interval-censored competing risks data is often required and plays an important role in many areas. For the situation, in addition to competing risk and interval censoring, another feature that makes the analysis difficult is that the failure cause may be unknown or missing. Most existing methods for addressing these challenges rely on two-stage estimation procedures, which could suffer efficiency loss and high computational cost. To overcome these, we propose a direct likelihood approach based on a mixture model framework. The proposed method accounts for both competing risks and missingness of event types directly in a likelihood function and facilitates estimation through a sieve maximum likelihood estimation, simplifying the estimation procedure and thus enhancing the estimation efficiency. The consistency and asymptotic normality of the resulting estimators are established, and the idea behind the proposed approach can be extended to other competing risks model frameworks. We demonstrate the promising performance of the proposed method in a comprehensive simulation study and illustrate its practical utility with an application to an Alzheimer’s disease study.

## 1. Introduction

Competing risks data arise when study subjects face potential multiple distinct and mutually exclusive causes of failure.^1,2^ This phenomenon is observable across various domains, encompassing cohort studies and clinical trials. For instance, in the study of dementia progression, the patients may not only contend with the risk of dementia-related outcomes but also confront the possibility of succumbing to non-dementia factors like heart attacks, leading to mortality. In this article, we will consider regression analysis of competing risks data in the presence of interval censoring and with missing causes of failure.

The analysis of competing risks data has garnered substantial attention due to the inherent limitations of the classical survival methods in dealing with multiple mutually exclusive causes of failure. With a pivotal emphasis on cumulative incidence functions, the cause-specific hazard model^3^ and the sub-distribution hazard approach^4^ come into prominence, where the latter directly models the cumulative incidence functions.^5–11^ Moreover, their suitability hinges on distinct research objectives.^12–14^ In addition, as discussed by Larson and Dinse^15^ and others, one can employ the mixture model approach that mirrors the classical cure model framework.^13,16–18^

In many studies, it is plausible that data concerning the precise cause of failure may not be accessible for all subjects who have experienced an event. In an Alzheimer’s disease (AD) study, for example, the death is attributed to a single underlying condition based on the reported death information, and the cause can be related to dementia, the cause of interest, or other causes. However, such information may not be completely documented due to lost of collection or the cause may be difficult for investigators to determine for some patients.^19^ A naive method of handling missing data problem is the complete case (CC) analysis from which all cases with missing information are excluded, and it is easy to see that this could potentially lead to biased estimates and erroneous inferences.^20,21^ Previous research has extensively examined the missing cause problem in competing risks data analysis; however, most studies have focused on right-censored outcomes. For example, Goetghebeur and Ryan^22^ and Craiu and Duchesne^23^ proposed cause-specific analyses using a semi-parametric proportional hazards model and an expectation–maximization (EM) algorithm-based inference method, respectively. Gao and Tsiatis^24^ developed augmented inverse probability weighted CC estimators, which were shown to be doubly robust. Bakoyannis et al.^25^ applied multiple imputation to the Fine-Gray model. A comprehensive overview of existing methods is provided by Bakoyannis et al.^26^ More recently, Lô et al.^27^ proposed a penalized likelihood estimation approach for cause-specific Cox models.

Noted that competing risks outcomes subject to interval censoring raise considerable complexities and hurdles to the inferential process. Recently several useful methods have been proposed in the literature to deal with missing cause for interval-censored competing risks data. For example, Mao et al.^28^ developed non-parametric maximum likelihood estimation implemented by an EM algorithm under a class of the semi-parametric transformation models to accommodate missing cause of failure, Do and Kim^29^ adopted the pseudo-value approach based on Rubin’s multiple imputations to address missing data in the estimation of cumulative incidence functions. Mitra et al.^30^ estimated cumulative incidences under the parametric Gompertz model assumption. Park et al.^31^ introduced an augmented inverse probability weighted sieve maximum likelihood estimator with doubly robust properties, using a two-stage estimation procedure based on a fitted model for missing probability. Guo et al.^32^ compared various methods, including inverse probability weighting and multiple imputation, for addressing the issue of missing event type.

In this article, we are interested in regression analysis of competing risks data in the presence of interval censoring when the causes of failure may be missing at random (MAR). This work was motivated by the data from AD neuroimaging initiative (ADNI), an extensive longitudinal multi-center study initiated in 2004 with the goal of developing biomarkers for the early detection and monitoring of AD. A crucial advancement in the study of AD revolves around the continuum from diagnosis to disease progression and, ultimately, mortality. However, the occurrence of death is influenced by not only the progression of dementia but also other related factors, such as heart attack or pneumonia, leading to competing events. Additionally, owing to the nature of periodic follow-ups or observations, the precise onset times of AD could only be ascertained to lie within intervals determined by two study visits. Furthermore, there was a subset of deceased individuals whose causes of death were missing possibly due to the absence of an autopsy or no records of the necessary information reported by their relatives.

For this problem, we adopt a mixture model approach and consider a semi-parametric mixture model, which has been widely used in biostatistical and epidemiological research^17,33^ and provides a general regression framework for the joint distribution of competing risks data.^15^ Building on the idea of Mao et al.,^28^ we propose an inference procedure that directly constructs the likelihood to handle missing failure causes. By incorporating information from all event types simultaneously through the mixture model, our approach improves estimation compared to existing methods. Unlike the two-stage augmented inverse probability approach,^31^ the proposed method employs a direct likelihood formulation, leading to more stable and reliable estimators in certain scenarios. To facilitate maximization of the likelihood, we apply a sieve approach based on Bernstein polynomials to address the computational challenges arising from the non-parametric baseline cumulative hazard function. This estimation approach offers computational advantages due to its straightforwardness and less complexity compared to the EM algorithm,^28^ which may suffer from slow convergence especially for a large dataset with substantial number of missing data, potentially leading to more stable optimization. Moreover, it avoids the integration required in cause-specific hazard models and inherently ensures the constraint that the sum of cumulative incidence functions (CIFs) remains below one, which is manually imposed in the subdistribution hazard model.

The rest of the article will be organized as follows. Section 2 will describe the proposed mixture model for competing risks data and present the proposed direct likelihood estimation method. Also in the section, the asymptotic properties of the resulting estimators will be established. In Section 3, we will present some results from a comprehensive simulation study conducted to assess the performance of the proposed method and they suggest that it works well in practical situations. Section 4 will discuss the application of the proposed method to the ADNI study described above and Section 5 concludes with a brief discussion. The theoretical proofs are provided in the Supplemental Material.

## 2. Proposed method

### 2.1. Data and model assumptions

Consider a study having K mutually exclusive types of competing events. Let $\left(T_{i},\;D_{i}\right)$ denote the event time and event type for subject i , where $D_{i}\in \{1,2,\ldots ,K\}$ and $D_{i}=k$ indicates that subject i experiences the k th type of event. We consider the common interval censoring scenario, where the event time $T_{i}$ is observed only within a sequence of monitoring times $0=V_{i,0}<V_{i,1}<\cdots <V_{i,J_{i}}$ , where $J_{i}$ is a random positive integer representing the number of the observation times. Define $V_{i}=\{V_{i,0},V_{i,1},\ldots ,V_{i,J_{i}}\}$ and let $\{L_{i},R_{i}\}\subset V_{i}$ be the smallest interval containing $T_{i}$ . The event indicator is defined as $\Delta_{i}=1$ if an event occurs during the study period (i.e. $T_{i}\leq V_{i,J_{i}}$ ), and $\Delta_{i}=0$ otherwise. We further define ${\tilde{\Delta }}_{i}=I(T_{i}\leq V_{i,1})$ and ${\bar{\Delta }}_{i}=\Delta_{i}-{\tilde{\Delta }}_{i}$ as the left- and interval-censoring indicators, respectively. For individuals experiencing the k th type of event, we define ${\tilde{\Delta }}_{ik}={\tilde{\Delta }}_{i}\;I(D_{i}=k)$ for left-censored cases and ${\bar{\Delta }}_{ik}={\bar{\Delta }}_{i}\;I(D_{i}=k)$ for interval-censored cases. Also, let $Z_{i}$ and $W_{i}$ be p - and q -dimensional covariate vectors associated with the failure processes of competing events and the occurrence probabilities of event types, respectively. Here $Z_{i}$ and $W_{i}$ could be the same, completely different, or share some common components. Throughout, we assume that $\left(T_{i},D_{i}\right)$ is conditionally independent of the observation process $V_{i}$ given the covariates. Notation without the subscript i represents the corresponding population analogues.

**Figure 1.**
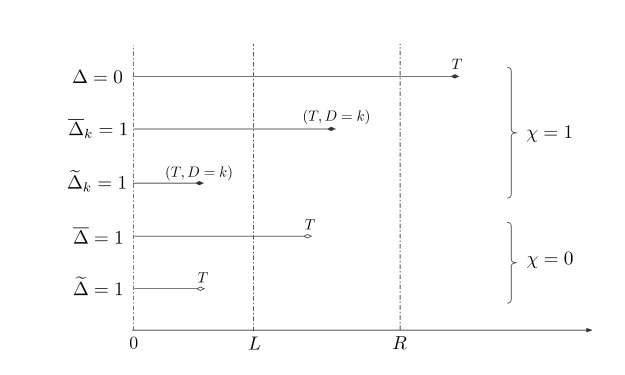
Illustration of interval-censored competing risks data with known cause of failure ( χ=1 ) and missing cause ( χ=0 ).

Let $O=\{O_{i}\;;\;i=1,\ldots ,n\}$ , where $O_{i}=(\;L_{i},\;R_{i},\;\Delta_{i},\;{\tilde{\Delta }}_{ik},\;{\bar{\Delta }}_{ik},\;Z_{i},\;W_{i}\;;\;k=1,\ldots ,K\;)$ be the observed data for individual i . Data from different individuals are supposed to be independent. In the presence of missing failure causes, we define a response (non-missingness) indicator $\chi_{i}=1$ for individual i if $\left\{{\tilde{\Delta }}_{ik},\;{\bar{\Delta }}_{ik}\right\}$ has been observed and $\chi_{i}=0$ otherwise. When right censoring occurs, we have $\Delta_{i}=0$ and thus ${\tilde{\Delta }}_{ik}={\bar{\Delta }}_{ik}=0$ . By definition, this results in $\chi_{i}=1$ . Figure 1 illustrates different scenarios in the interval-censored competing risks data with and without missing failure causes. In this case, the observed data for individual i is denoted by

$$O_{i}=\left\{ \begin{array}{rlr} & (\;L_{i},\;R_{i},\;\Delta_{i},\;{\tilde{\Delta }}_{ik},\;{\bar{\Delta }}_{ik},\;Z_{i},\;W_{i}\;;\;k=1,\ldots ,K\;), & \text{ if }\chi_{i}=1\;\\ & (\;L_{i},\;R_{i},\;\Delta_{i},\;Z_{i},\;W_{i}\;;\;k=1,\ldots ,K\;), & \text{ if }\chi_{i}=0 \end{array}\right.$$

We assume that the missing mechanism follows the MAR principle defined by Rubin,^34^ meaning that the probability of missing depends on the observed value not the missing value. That is, the probability of a failure cause being missed given the failure time and covariates does not depend on the cause itself. In other words, for individual i with $\Delta_{i}=1$ , we assume

$$P(\chi_{i}=1|\Delta_{i}=1,\;{\tilde{\Delta }}_{ik},\;{\bar{\Delta }}_{ik},\;L_{i},\;R_{i},\;Z_{i},\;W_{i}\;;\;k=1,\ldots ,K\;;\;\zeta )=P(\chi_{i}=1|\Delta_{i}=1,\;L_{i},\;R_{i},\;Z_{i},\;W_{i}\;;\;\zeta )$$

where ζ is a vector of the associated parameters. Note that if (a) the missing data mechanism is MAR and (b) parameters in the failure time model and ζ are distinct in the sense that their joint parameter space is the product of the two individual parameter spaces, the missing data mechanism is said to be ignorable.^35^ Since assumption (b) is almost always true in real-world settings, ignorability and MAR (together with missing completely at random) are sometimes viewed as equivalent.^36^ Under these assumptions, one can effectively proceed with the analysis without addressing the missingness about the cause of failure. It is worth noting that the above assumptions also imply that $\chi_{i}$ and $D_{i}$ are independent given $L_{i}$ , $R_{i}$ , $Z_{i}$ , and $W_{i}$ for $\Delta_{i}=1$ . That is,

$$\begin{array}{rl} P(D_{i}=k|\chi_{i}=1,\;L_{i},\;R_{i},\;Z_{i},\;W_{i}) & =P(D_{i}=k|\chi_{i}=0,\;L_{i},\;R_{i},\;Z_{i},\;W_{i})=P(D_{i}=k|L_{i},\;R_{i},\;Z_{i},\;W_{i}) \end{array}$$

for k=1,…,K .

For inference, as mentioned above, we will adopt the mixture model approach and assume that T can be written as follows:

$$T=\sum_{k=1}^{K}T_{k}^{*}\times I\;(D=k)\;$$

with $T_{k}^{*}$ being the failure time from event type k . Given the cause of failure D=k , we will assume that the covariate effects can be described by the following Cox proportional hazards model^37,38^ in terms of the survival function

(1) $$S_{k}(t;\;\beta_{k},\Lambda_{k})=P(T_{k}^{*}\geq t|D=k,\;Z)=\exp\;\{-\Lambda_{k}(t)\;\exp\;(\beta_{k}^{⊤}Z)\},\;\;k=1,\ldots ,K$$

where $\Lambda_{k}(\cdot )$ is an unspecified and strictly increasing nonnegative function and $\beta_{k}$ is the vector of regression coefficients. Model (1) particularly describes the cause-specific conditional survival function of $T_{k}^{*}$ given the occurrence of failure cause k and covariates.

To model the relationship between the marginal probability of experiencing failure from cause k and the covariate W , we postulate a multinomial logistic regression

(2) $$\log\;\left\{\frac{P(\;D=k|W\;)}{P(\;D=1|W\;)}\right\}=\gamma_{k}^{⊤}\;\tilde{W},\;\;k=2,\ldots ,K$$

where $\tilde{W}=(1,\;W^{⊤}{)}^{⊤}$ and $\gamma_{k}$ is an unknown parameter vector. The equation in (2) can be rewritten as follows:

$$\pi_{k}\;(\gamma ):=P(\;D=k|W\;)=\frac{\exp\;(\gamma_{k}^{⊤}\;\tilde{W})}{\sum_{j=1}^{K}\exp\;(\gamma_{j}^{⊤}\;\tilde{W})},\;\;k=1,\ldots ,K$$

with $\gamma_{1}=(0,\ldots ,0{)}^{⊤}$ . Let $\gamma \;=\;(\gamma_{2},\ldots ,\gamma_{K}{)}^{⊤}$ . By models (1) and (2), the overall survival function for T can then be expressed in the form of a finite mixture model

(3) $$S(t;\;\theta )=P(T\geq t|Z,W)=\sum_{k=1}^{K}\pi_{k}(\gamma )\;S_{k}(t;\;\beta_{k},\Lambda_{k})$$

The mixture approach described above provides a joint framework that simultaneously accounts for the event time T and event type D , while allowing for competing events to be dependent through shared covariates. The multinomial logistic regression component models the marginal probabilities of different event types, and the proportional hazards component models the distribution of event time conditional on the event type. This structure offers a flexible way to capture the dependence between competing risks. By conditioning on the event type, we can separately estimate the effects of covariates on event type probabilities and event time distributions. This separation is computationally tractable and allows for meaningful parameter interpretation.^33^ Specifically, parameters in the multinomial logistic regression describe how covariates influence the relative risk of different event types, whereas those in the proportional hazards model characterize the effect of covariates on the event time for a given event type. Furthermore, under the mixture model, the constraint $\sum_{k}\pi_{k}=1$ inherently ensures that the sum of the cumulative incidence functions over all failure causes equals one. It is noted that this constraint may be violated in the subdistribution model,^4^ resulting in an invalid model when a joint analysis is of interest in the competing risks setting.^17^ In the following, we will focus on the estimation of parameters $\theta =\{\beta_{k},\;\Lambda_{k},\;\gamma ;\;k=1,\ldots ,K\}$ in model (3) while taking into account the missing information on the cause of failure.

### 2.2. Estimation procedure

An advantage of the mixture model in (3) is its relative ease in summarizing the cumulative incidence rate for each competing cause. Under it, the CIF corresponding to the k th event can be obtained by the following equation:

(4) $$F_{k}(t;\;\theta )=P(T\leq t,D=k)=P(T\leq t|Z,\;D=k)\;P(D=k|W)=\pi_{k}(\gamma )\{1-S_{k}(t;\;\beta_{k},\Lambda_{k})\}$$

In the presence of missing cause, we present the likelihood function of θ by considering three distinct cases of failure outcomes according to censoring type and missing status. For the sake of simplicity in notation, we suppress the dependence on covariates in the following. Based on model (3) and the result in (4), the likelihood contribution of a subject in each case is given as follows:

Case 1. Right censoring: if subject i has $\Delta_{i}=0$ , it indicates that the individual did not experience any event by the end of the study, corresponding to the first sample shown in Figure 1. So, subject i ’s contribution to the likelihood is $S(R_{i})$ .
Case 2. Left/interval censoring and observed cause: if subject i has $\Delta_{i}=1$ and $\chi_{i}=1$ , which indicates that the subject has been experienced event k (i.e. ${\tilde{\Delta }}_{ik}=1$ or ${\bar{\Delta }}_{ik}=1$ ), corresponding to the second and the third samples in Figure 1. In this case, subject i contributes
$$\{F_{k}(L_{i}){\}}^{{\tilde{\Delta }}_{ik}}\{F_{k}(R_{i})-F_{k}(L_{i}){\}}^{{\bar{\Delta }}_{ik}}P(\chi_{i}=1|\Delta_{i}=1,\;L_{i},\;R_{i})$$
  to the likelihood.
Case 3. Left/interval censoring but missing cause: if subject i has $\Delta_{i}=1$ and $\chi_{i}=0$ , whose failure time is observed but with missed the failure cause, corresponding to the last two samples shown in Figure 1. The subject then contributes
$$\{1-S(L_{i}){\}}^{{\tilde{\Delta }}_{i}}\{S(L_{i})-S(R_{i}){\}}^{{\bar{\Delta }}_{i}}P(\chi_{i}=0|\Delta_{i}=1,\;L_{i},\;R_{i})$$

Combining the above three cases with the assumptions of independent censoring, the MAR and ignorability, the full likelihood of θ based on the observed data can be obtained by thefollowing equation:

(5) $$\begin{array}{rl} L(\theta ) & =\prod_{i=1}^{n}\prod_{k=1}^{K}[\{F_{k}(L_{i}){\}}^{{\tilde{\Delta }}_{ik}}\{F_{k}(R_{i})-F_{k}(L_{i}){\}}^{{\bar{\Delta }}_{ik}}{]}^{\Delta_{i}\;\chi_{i}}\\ & \;\times [\{1-S(L_{i}){\}}^{{\tilde{\Delta }}_{i}}\{S(L_{i})-S(R_{i}){\}}^{{\bar{\Delta }}_{i}}{]}^{\Delta_{i}\;(1-\chi_{i})}\{S(R_{i}){\}}^{1-\Delta_{i}} \end{array}$$

For estimation of θ , it is apparent that a natural approach would be to directly maximize the above likelihood function (5) or its logarithm but this is clearly difficult due to the presence of non-parametric baseline cumulative hazard functions $\Lambda_{k}(\cdot )$ . To address this, we propose to employ the sieve approach described below by following Zhou et al.,^39^ which approximates the unknown functions by using the Bernstein polynomials before the maximization. Define the parameter space

$$\Theta =\{\theta =(\beta_{k},\;\gamma ,\;\Lambda_{k};\;k=1,\ldots ,K)\in B⊗\{{⊗}_{k=1}^{K}M^{k}\}\}$$

where $B=\{\eta =\{\beta_{k},\gamma ;\;k=1,\ldots ,K\}\in R^{Kp+(K-1)(q+1)}:||\eta ||\leq M\}$ with M being a positive constant and $M^{k}$ is the collection of all bounded and continuous nondecreasing, nonnegative functions over the interval $\left[t_{l},t_{r}\right]$ with $0\leq t_{l}<t_{r}<\infty$ . In practice, as in the numerical study below, one could take $t_{l}$ and $t_{r}$ to be the smallest and the largest values of the $L_{i}$ ’s and $R_{i}$ ’s, respectively. Also define the sieve space

(6) $$\Theta_{n}=\{\theta_{n}=(\eta ,\;\Lambda_{kn};\;k=1,\ldots ,K)\in B⊗\{{⊗}_{k=1}^{K}M_{n}^{k}\}\}$$

where

$$\begin{array}{r} M_{n}^{k}=\{\Lambda_{kn}(t)=\sum_{j=0}^{m}\psi_{k}^{j}\;B_{j}(t,m,t_{l},t_{r}):0\leq \psi_{k}^{0}\leq \psi_{k}^{1}\leq \cdots \leq \psi_{k}^{m},\sum_{0\leq j\leq m}|\psi_{k}^{j}|\leq M_{n}\} \end{array}$$

for k=1,…,K , where $M_{n}$ ensures that the $\Lambda_{kn}$ is bounded, and

Bj(t,m,tl,tr)=(mj)(t−tltr−tl)j×(1−t−tltr−tl)m−j,j=0,…,m

with the degree $m=o(n^{\nu })$ for some ν∈(0,1) . The sieve maximum likelihood estimator for θ is then defined as the value of θ that maximizes the log-likelihood function ℓ(θ)=logL(θ) over $\Theta_{n}$ , denoted by $\hat{\theta }=\{\hat{\eta },\;{\hat{\Lambda }}_{k};\;k=1,\ldots ,K\}$ .

Note that the above estimation approach requires some restrictions to ensure the nonnegativity and monotonicity of the functions $\Lambda_{k}(\cdot )$ , k=1,…,K . However, these restrictions can be readily removed by employing suitable reparameterization techniques. For this, similar to Lou et al.,^10^ we reparameterize the coefficients by defining them in terms of a set of unconstrained parameters $\left\{{\tilde{\psi }}_{k}^{0},\ldots ,{\tilde{\psi }}_{k}^{m}\right\}$ as $\psi_{k}^{0}=\exp\;({\tilde{\psi }}_{k}^{0})$ and $\psi_{k}^{j}=\psi_{k}^{j-1}+\exp\;({\tilde{\psi }}_{k}^{j})$ for j=1,…,m . This approach ensures that the monotonicity and nonnegativity constraints on $\left\{\psi_{k}^{j}\right\}$ are automatically satisfied, allowing for unconstrained optimization with respect to $\left\{{\tilde{\psi }}_{k}^{j}\right\}$ . It is worth noting that the utilization of the sieve approach transfers an estimation problem involving both finite and infinite-dimensional parameters into a relatively simpler estimation problem involving only finite-dimensional parameters. In other words, it has the advantage of significantly reducing the dimensionality of the optimization problem, thus lowering the computational burden. Regarding the choice of approximation functions, besides Bernstein polynomials, other options such as B-splines and piecewise linear functions^8,26^ can also be used. An advantage of Bernstein polynomials is that they can naturally model the nonnegativity and monotonicity of $\Lambda_{k}$ ’s with simple restrictions that can be easily removed through reparameterization in computation.^40^ It also has the optimal shape-preserving property among all approximation polynomials.^41^ Furthermore, compared to the existing methods,^8,26^ they are easier to work with since they do not require the specification of interior knots. To obtain the estimator $\hat{\theta }$ by maximizing ℓ(θ) , various existing optimization methods can be utilized. We use the Matlab function fminunc in our numerical studies. Alternatively, one can also use R optimization packages for this purpose. In addition, we have the following two remarks.

Remark 1 For the implementation of the estimation procedure proposed, one needs to specify m or v . The most common choice of v is 1/4 in practice.^10^ As illustrated in the numerical studies below, we use m=3 to align with $n^{0.25}$ for n=200 or 400 . In general, the proposed method is not sensitive to the choice of m , and results are typically robust for the values in a neighborhood of $n^{0.25}$ . When additional tuning is desired, one may select m by a simple grid search over a small candidate set,^39^ or by an information criterion such as AIC.^42^

Remark 2 It is worth noting that the proposed likelihood contribution idea under the MAR assumption can be extended to various competing risks models, including the cause-specific hazard model and the Fine-Gray model, as considered by Li^8^ and Bakoyannis et al.,^43^ respectively. A related approach employing an EM algorithm for the subdistribution hazard model is presented by Mao et al.^28^ More details are provided in the Appendix.

### 2.3. Asymptotic properties

To establish the asymptotic properties of the proposed estimator $\hat{\theta }$ , let $\theta_{0}=(\eta_{0},\Lambda_{k0};\;k=1,\ldots ,K)$ be the true value of θ and ||v|| be the Euclidean norm for a vector v . We define the $L_{2}$ -metric for the distance between two parameters $\theta^{\left(1\right)}=(\eta^{\left(1\right)},\;\Lambda_{k}^{\left(1\right)};\;k=1,\ldots ,K)\in \Theta$ and $\theta^{\left(2\right)}=(\eta^{\left(2\right)},\;\Lambda_{k}^{\left(2\right)};\;k=1,\ldots ,K)\in \Theta$ by

$$d(\theta^{\left(1\right)},\theta^{\left(2\right)})=\{||\eta^{\left(1\right)}-\eta^{\left(2\right)}|{|}^{2}+\sum_{k=1}^{K}||\Lambda_{k}^{\left(1\right)}-\Lambda_{k}^{\left(2\right)}|{|}_{2}^{2}{\}}^{1/2}$$

where

$$||\Lambda_{k}^{\left(1\right)}-\Lambda_{k}^{\left(2\right)}|{|}_{2}^{2}=E[\Lambda_{k}^{\left(1\right)}(L)-\Lambda_{k}^{\left(2\right)}(L){]}^{2}+E[\Lambda_{k}^{\left(1\right)}(R)-\Lambda_{k}^{\left(2\right)}(R){]}^{2},\;k=1,\ldots ,\;K$$

The following theorems give the asymptotic consistency and normality of the proposed estimators under the regularity conditions given in the Supplemental Material.

Theorem 1 Assume that Conditions 1–4 given in the Supplemental Material hold. Then we have that $d(\hat{\theta },\theta_{0})\to 0$ in probability.

Theorem 2 Assume that Conditions 1–4 given in the Supplemental Material hold. Then it can be shown that $d(\hat{\theta },\theta_{0})=O_{p}(n^{-\min\{(1-\nu )/2,\nu r/2\}})$ where 0<ν<1/2 such that $m=o(n^{\nu })$ and r is defined in Condition 3.

Theorem 3 Assume that Conditions 1–4 given in the Supplemental Material hold with r>2 in Condition 3 and ν>1/(2r) . Then it can be shown that $\sqrt{n}\;(\hat{\eta }-\eta_{0})$ converges in distribution to a zero-mean multivariate normal distribution as n→∞ and the estimator $\hat{\eta }$ is asymptotically efficient.

The proofs of the results above are sketched in the Supplemental Material. Note that from Theorem 2 that the choice of ν=1/(1+r) yields the optimal convergence rate $n^{r/(2(1+r))}$ , which equals $n^{1/3}$ when r=2 and improves monotonically as r increases. Moreover, Theorem 3 implies that the asymptotic variance of $\hat{\eta }$ attains the semi-parametric efficiency bound. For inference, it is apparent that we need to estimate the covariance matrix of $\hat{\eta }$ and for this, a natural approach would be to directly estimate the asymptotic covariance matrix of $\hat{\eta }$ . On the other hand, this would be difficult since one can see from the proof of Theorem 3 that it has a complicated analytical form. For this, one approach is to employ the profile likelihood function method,^44,45^ and the other is to base it on the inverse of the Hessian matrix of the log-likelihood function ℓ(θ) .^39^ In what follows, we adopt variance estimation via the inverse observed information. After the sieve approximation, the log-likelihood ℓ(θ) depends only on a finite-dimensional parameter vector. We maximize ℓ(θ) using a quasi-Newton algorithm to obtain $\hat{\theta }$ . The asymptotic covariance matrix is then computed in a single, non-iterative step by inverting the negative Hessian of ℓ(θ) , evaluated numerically at $\hat{\theta }$ . The numerical study below indicates that this approach performs well in practice. When the Hessian is difficult to evaluate or exhibits numerical instability, a non-parametric bootstrap offers a robust alternative for variance estimation, as demonstrated by its satisfactory performance in the numerical study below. In terms of the number of bootstrap resamples, a practical strategy is to monitor the stability of standard errors as the resample size increases and stop when these quantities stabilize, thereby controlling computation while maintaining accuracy.

## 3. Simulation study

In this section, we present some results obtained from a simulation study conducted to assess the finite sample performance of the proposed estimation procedure. In the study, it was assumed that there existed two event types, D=1 or 2 , and two covariates $Z_{1}$ and $Z_{2}$ following the uniform distribution U(−2,2) and the Bernoulli distribution with probability 0.6, respectively. Given the covariates, the competing risks data (T,D) were generated with $\Lambda_{1}(t)=t$ and $\Lambda_{2}(t)=2.5t^{2}$ and the true values of the regression parameters being $\beta_{1}=(\beta_{1,1},\;\beta_{1,2}{)}^{⊤}=(-0.3,0.5{)}^{⊤}$ , $\beta_{2}=(\beta_{2,1},\;\beta_{2,2}{)}^{⊤}=(0.5,-0.3{)}^{⊤}$ , and $\gamma =(\gamma_{0},\;\gamma_{1},\;\gamma_{2}{)}^{⊤}=(0.1,0.4,-0.3{)}^{⊤}$ .

To generate observation times and the corresponding censoring intervals, following Bakoyannis et al.^43^ and Park et al.,^31^ we simulated a sequence of visit times from an exponential distribution with hazard rate 3 and a maximum study period of 3. This design yields an overall censoring rate of ∼ 8% and an average inter-visit gap of about 0.25. Then, if all observation time points were larger than T , L was set to be the smallest observation time point, and R was set to be the time point immediately following the smallest one. If all observation time points were smaller than T , L was taken as the second largest observation time point, and R was set to be the largest observation time point. Otherwise, L was assigned as the largest observation time point that was smaller than T , and R was set to be the smallest observation time point that was larger than T . Furthermore, the missing indicator was generated based on $P(\chi =1|O)=1/\{1+\exp\;(\xi_{0}+\xi_{1}^{⊤}Z)\}$ with $\xi_{1}=(-0.5,0.3{)}^{⊤}$ , and $\xi_{0}=-0.50$ or 0.20 , giving the missing rate (MR) around 40% or 60% , respectively. The results given below are based on 1000 replications, n=200 or 400 , and m=3 for Bernstein polynomials.

**Figure 2.**
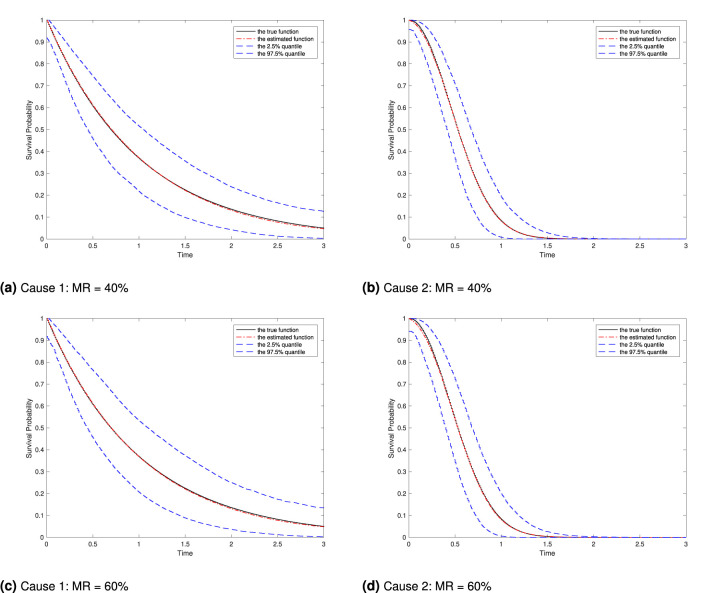
Estimated baseline survival curves along with the true curve regarding Table 1 with n=200 : the true function (solid), the estimated function (dash-dot), and the 2.5% quantile and 97.5% quantiles (dashed). (a) Cause 1: MR = 40%, (b) Cause 2: MR = 40%, (c) Cause 1: MR = 60%, and (d) Cause 2: MR = 60%.

Table 1 presents the results on estimation of regression parameters given by the proposed method and they include the estimated bias (Bias) given by the average of the estimates minus the true value, the sample standard deviation (SD) of the estimates, the average of the estimated standard errors (ESEs) based on the inverse of the Hessian matrix, and the 95% empirical coverage probability (CP). The results indicate that the proposed estimator appears to be unbiased with increased accuracy and reduced variation as the sample size increases. In addition, the CP results are close to the 95% nominal level, suggesting that the normal approximation to the distribution of the proposed estimator is appropriate. Figure 2 presents the estimated baseline survival functions along with the 2.5% and 97.5% quantiles of the empirical distribution of the estimates for both event types under the two settings described in Table 1, with n=200 . The average of the estimated baseline survival curves exhibit close alignment with their corresponding true values in both settings, indicating the promising performance of the proposed Bernstein polynomial approximation.

**Table 1.** Simulation results based on the proposed method.

		n=200				n=400			
MR	Param	Bias	SD	ESE	CP	Bias	SD	ESE	CP
40%	$\beta_{1,1}$	−0.0250	0.1336	0.1232	0.947	−0.0133	0.0862	0.0837	0.947
	$\beta_{1,2}$	0.0313	0.2771	0.2538	0.938	0.0125	0.1806	0.1738	0.946
	$\beta_{2,1}$	0.0296	0.1499	0.1380	0.932	0.0183	0.0977	0.0955	0.948
	$\beta_{2,2}$	−0.0309	0.3134	0.2844	0.933	−0.0191	0.2121	0.1978	0.933
	$\gamma_{0}$	−0.0209	0.2916	0.2911	0.955	0.0099	0.2045	0.2028	0.956
	$\gamma_{1}$	0.0180	0.1766	0.1689	0.944	0.0053	0.1213	0.1173	0.947
	$\gamma_{2}$	0.0068	0.3636	0.3779	0.970	−0.0056	0.2630	0.2632	0.955
60%	$\beta_{1,1}$	−0.0320	0.1479	0.1324	0.933	−0.0155	0.0935	0.0897	0.951
	$\beta_{1,2}$	0.0253	0.2959	0.2661	0.943	0.0095	0.1907	0.1819	0.945
	$\beta_{2,1}$	0.0418	0.1696	0.1513	0.929	0.0280	0.1129	0.1046	0.942
	$\beta_{2,2}$	−0.0503	0.3371	0.3074	0.937	−0.0214	0.2199	0.2130	0.943
	$\gamma_{0}$	−0.0270	0.3354	0.3331	0.957	−0.0050	0.2349	0.2324	0.950
	$\gamma_{1}$	0.0311	0.2039	0.1946	0.944	0.0126	0.1411	0.1344	0.949
	$\gamma_{2}$	0.0068	0.4424	0.4301	0.954	0.0047	0.3050	0.2990	0.944

MR: missing rate; Param: parameter; Bias: estimated bias; SD: standard deviation; ESE: estimated standard error; CP: coverage probability.

We also conducted simulations to compare the proposed approach with the naive CC approach, which involved analyzing only subjects with complete information. The results are presented in Table 2, where we report the estimated regression coefficients for the CCs, along with the results replicated from Table 1 with n=200 . To facilitate ease of comparison, the estimated mean squared error (MSE) has also been included in the table. Compared to the CC approach, the proposed method exhibits higher efficiency in estimating regression coefficients in each component of the mixture model. In addition, the method demonstrates notable improvements in parameter estimation accuracy.

**Table 2.** Simulation results based on the proposed method and the complete case analysis with n=200 .

		Proposed method					Complete case analysis				
MR	Param	Bias	SD	ESE	CP	MSE	Bias	SD	ESE	CP	MSE
40%	$\beta_{1,1}$	−0.0250	0.1336	0.1232	0.947	0.1359	−0.0241	0.1643	0.1495	0.936	0.1661
	$\beta_{1,2}$	0.0313	0.2771	0.2538	0.938	0.2789	0.0701	0.3305	0.3026	0.928	0.3379
	$\beta_{2,1}$	0.0296	0.1499	0.1380	0.932	0.1528	0.0518	0.1847	0.1663	0.932	0.1918
	$\beta_{2,2}$	−0.0309	0.3134	0.2844	0.933	0.3149	−0.0427	0.3736	0.3441	0.942	0.3760
	$\gamma_{0}$	−0.0209	0.2916	0.2911	0.955	0.2923	0.0233	0.3034	0.3028	0.955	0.3043
	$\gamma_{1}$	0.0180	0.1766	0.1689	0.944	0.1775	0.0354	0.1874	0.1812	0.950	0.1907
	$\gamma_{2}$	0.0068	0.3636	0.3779	0.970	0.3637	−0.0345	0.3820	0.3967	0.959	0.3836
60%	$\beta_{1,1}$	−0.0320	0.1479	0.1324	0.933	0.1513	−0.0345	0.2122	0.1827	0.925	0.2150
	$\beta_{1,2}$	0.0253	0.2959	0.2661	0.943	0.2970	0.1017	0.4044	0.3572	0.930	0.4170
	$\beta_{2,1}$	0.0418	0.1696	0.1513	0.929	0.1747	0.0702	0.2322	0.2060	0.923	0.2426
	$\beta_{2,2}$	−0.0503	0.3371	0.3074	0.937	0.3408	−0.0795	0.4744	0.4246	0.933	0.4810
	$\gamma_{0}$	−0.0270	0.3354	0.3331	0.957	0.3365	0.0549	0.3551	0.3564	0.958	0.3593
	$\gamma_{1}$	0.0311	0.2039	0.1946	0.944	0.2063	0.0578	0.2260	0.2194	0.946	0.2333
	$\gamma_{2}$	0.0068	0.4424	0.4301	0.954	0.4425	−0.0543	0.4809	0.4681	0.951	0.4840

MR: missing rate; Param: parameter; Bias: estimated bias; SD: standard deviation; ESE: estimated standard error; CP: coverage probability.

In the preceding study, we set m=3 . As noted in Remark 1 above, the proposed method is expected to be robust to the choice of m . To assess this, we replicated the study yielding Table 1 for n=400 except using alternative sieve dimensions m∈{2,4,6} , and the results are presented in Table 3. They indicate that the estimator is indeed insensitive to moderate changes in m , with performance remaining stable across these choices. We also examined alternative visit schedules and right-censoring rates, and the corresponding results are provided in the Supplemental Material and indicate that the proposed method performs well under these settings. In addition, although our implementation employs Bernstein polynomials for sieve approximation, alternative bases may be used. To assess the sensitivity to the choice of basis, we replaced the Bernstein polynomials with B-splines and obtained similar results in the Supplemental Material, further suggesting the robustness of the method.

**Table 3.** Simulation results under different choices of m with n=400 .

		MR = 40%				MR = 60%			
m	Param	Bias	SD	ESE	CP	Bias	SD	ESE	CP
2	$\beta_{1,1}$	−0.0123	0.0923	0.0896	0.953	−0.0079	0.0856	0.0843	0.951
	$\beta_{1,2}$	0.0131	0.1922	0.1818	0.942	0.0037	0.1730	0.1741	0.956
	$\beta_{2,1}$	0.0014	0.0898	0.0908	0.950	−0.0056	0.0870	0.0848	0.939
	$\beta_{1,2}$	0.0053	0.2091	0.2048	0.952	0.0012	0.1955	0.1908	0.935
	$\gamma_{0}$	−0.0054	0.2198	0.2310	0.972	−0.0089	0.2044	0.2027	0.946
	$\gamma_{1}$	0.0095	0.1369	0.1332	0.953	0.0195	0.1214	0.1175	0.946
	$\gamma_{2}$	−0.0113	0.3020	0.2991	0.948	0.0050	0.2576	0.2641	0.955
4	$\beta_{1,1}$	−0.0191	0.0916	0.0896	0.945	−0.0133	0.0875	0.0846	0.945
	$\beta_{1,2}$	0.0077	0.1858	0.1812	0.950	0.0075	0.1812	0.1749	0.939
	$\beta_{2,1}$	0.0253	0.1075	0.1045	0.945	0.0214	0.0975	0.0954	0.942
	$\beta_{1,2}$	−0.0085	0.2178	0.2116	0.943	−0.0197	0.2044	0.1959	0.948
	$\gamma_{0}$	0.0010	0.2269	0.2312	0.950	−0.0065	0.2075	0.2026	0.948
	$\gamma_{1}$	0.0078	0.1413	0.1342	0.943	0.0031	0.1195	0.1117	0.947
	$\gamma_{2}$	−0.0016	0.3055	0.2981	0.940	−0.0004	0.2747	0.2633	0.939
6	$\beta_{1,1}$	−0.0208	0.0955	0.0893	0.947	−0.0161	0.0897	0.0843	0.942
	$\beta_{1,2}$	0.0284	0.1832	0.1821	0.944	0.0166	0.1783	0.1747	0.953
	$\beta_{2,1}$	0.0206	0.1136	0.1047	0.938	0.0213	0.0987	0.0956	0.946
	$\beta_{1,2}$	−0.0117	0.2320	0.2123	0.936	−0.0207	0.1968	0.1962	0.949
	$\gamma_{0}$	−0.0107	0.2480	0.2327	0.937	−0.0050	0.2018	0.2030	0.952
	$\gamma_{1}$	0.0090	0.1360	0.1229	0.954	0.0114	0.1186	0.1172	0.950
	$\gamma_{2}$	0.0169	0.3085	0.2986	0.947	−0.0016	0.2629	0.2635	0.947

MR: missing rate; Param: parameter; Bias: estimated bias; SD: standard deviation; ESE: estimated standard error; CP: coverage probability.

Next, we evaluated the proposed estimator under a missing-not-at-random (MNAR) mechanism, noting that the method is developed under the MAR assumption. We retained the settings of Table 1 and modified the missingness model to $P(\chi =1|O)\;=\;1/\;[1+\exp\;\{\;0.20+\xi_{1}^{⊤}Z-\xi_{D}\;I(D=1)\;\}]$ . When $\xi_{D}\neq 0$ , the probability of observation depends on the (unobserved) event type, inducing MNAR. We varied $\xi_{D}$ to assess the sensitivity to deviations from MAR. Table 4 presentes the results for the regression parameters β in the cause-specific components with n=400 . From the table, we observe that the estimator shows reasonable robustness under mild deviations from the MAR assumption, that is, for small values of $\xi_{D}$ , the bias and coverage probabilities remain acceptable. However, as the violation of MAR becomes more severe (larger $\xi_{D}$ ), the performance deteriorates, reflected in increasing bias and declining CP. This is expected since the missingness mechanism directly depends on the unobserved failure type, making it non-ignorable and thus violating the key assumptions of the proposed model.

**Table 4.** Simulation results based on the proposed method with missing cause under MNAR and n=400 .

Param	Bias	SD	ESE	CP	Bias	SD	ESE	CP
	$\xi_{D}=0.25$				$\xi_{D}=0.50$			
$\beta_{1,1}$	0.0018	0.0887	0.0851	0.945	0.0100	0.0850	0.0814	0.939
$\beta_{1,2}$	0.0000	0.1783	0.1747	0.948	−0.0191	0.1796	0.1694	0.942
$\beta_{2,1}$	0.0402	0.1137	0.1057	0.931	0.0589	0.1146	0.1079	0.924
$\beta_{1,2}$	−0.0425	0.2296	0.2169	0.931	−0.0449	0.2224	0.2202	0.944
	$\xi_{D}=0.75$				$\xi_{D}=1.00$			
$\beta_{1,1}$	0.0280	0.0845	0.0781	0.906	0.0279	0.0813	0.0762	0.909
$\beta_{1,2}$	−0.0317	0.1661	0.1639	0.939	−0.0444	0.1674	0.1604	0.929
$\beta_{2,1}$	0.0752	0.1186	0.1100	0.914	0.0927	0.1242	0.1115	0.879
$\beta_{1,2}$	−0.0756	0.2476	0.2262	0.922	−0.0671	0.2460	0.2285	0.940

MNAR: missing-not-at-random; Param: parameter; Bias: estimated bias; SD: standard deviation; ESE: estimated standard error; CP: coverage probability.

As discussed in Remark 2, the proposed method can be readily extended to other model frameworks for competing risks data. To see this numerically, we repeated the study above by replacing the mixture model with the semi-parametric transformation models. Similar to the settings by Park et al.,^31^ the competing risks data were generated under sub-distributional proportional odds models with $\Lambda_{1}(t)=0.4\{1-\exp\;(-0.6\;t)/0.6\}$ and $\Lambda_{2}(t)=0.75\{1-\exp\;(-0.5t)/0.5\}$ , along with two covariates of interest, $Z_{1}$ and $Z_{2}$ , where $Z_{1}$ was simulated from the Bernoulli distribution with a probability of 0.4, and $Z_{2}$ was simulated from the standard normal distribution. The values for the regression coefficients were $\beta_{1}=(\beta_{1,1},\beta_{1,2}{)}^{⊤}=(0.5,-0.3{)}^{⊤}$ , $\beta_{2}=(\beta_{2,1},\beta_{2,2}{)}^{⊤}=(-0.5,0.3{)}^{⊤}$ . Observation time points and interval censoring were generated as before. The probability of nonmissingness was assumed to be $\text{logit}\{P(\chi =1|O)\}=\xi_{0}-0.5\;Z_{1}+0.6\;Z_{2}$ , where $\xi_{0}$ takes values 0.4 , −0.7 , and −1.9 to achieve pre-specified missingness rates 40%, 60%, and 80%, respectively.

Instead of the proposed method, one may also apply the two-stage augmented inverse probability weighted (AIPW) estimation. The related approach is presented by Park et al.,^31^ which assumes a weaker MAR assumption by incorporating auxiliary variables into the observed data. To maintain consistency, we focus on the scenario where no auxiliary variables are available. To compare the proposed method with the two-stage AIPW approach, Table 5 presents the estimation results from both methods based on 400 samples with 1000 replications, where variance estimation in this comparative study was facilitated using a non-parametric bootstrap with 100 resamples. Although Hessian-based (observed information) estimators are computationally efficient, the bootstrap offers a robust and flexible alternative, especially in the presence of complex constraints that can render direct Hessian evaluation unstable or impractical.^10^ Specifically, the ESE was calculated based on 100 bootstrap samples and the standard deviation of $\hat{\beta }$ was estimated using the median absolute deviation, namely, 1.4826×MAD , where $\mathrm{MAD}=\mathrm{median}\{|{\hat{\beta }}^{\left(b\right)}-\mathrm{median}({\hat{\beta }}^{\left(b\right)})|\}$ with ${\hat{\beta }}^{\left(b\right)}$ representing the estimated values obtained from the b th bootstrap sample^46^ for b=1,…,100 . One can see from the table that the proposed method works reasonably well even under the general semi-parametric transformation model framework. The bootstrapping standard errors are close to the sample standard deviations and confidence intervals have CPs close to the 95% nominal level. Compared with the two-stage AIPW, our proposed estimator exhibits comparable or even smaller bias and consistently lower MSE. When the MR grows up to 80%, the efficiency gains in the proposed estimation tend to be more significant, as evidenced by considerable increases in the relative efficiency (RE), calculated as the ratio of SD ${}^{2}$ of the AIPW estimates to SD ${}^{2}$ of the proposed estimates. This might be attributed to the poor estimation of logistic regression required in the first stage of the AIPW approach with unbalanced data.

**Table 5.** Simulation results based on the proposed method under the semi-parametric transformation model and the two-stage AIPW method with n=400 .

		Proposed method					AIPW					
MR	Param	Bias	SD	ESE	CP	MSE	Bias	SD	ESE	CP	MSE	RE
40%	$\beta_{1,1}$	0.0005	0.2558	0.2681	0.956	0.2558	−0.0008	0.2665	0.2782	0.957	0.2665	1.0854
	$\beta_{1,2}$	−0.0021	0.1323	0.1315	0.941	0.1323	−0.0010	0.1468	0.1426	0.942	0.1468	1.2312
	$\beta_{2,1}$	−0.0065	0.2537	0.2663	0.951	0.2538	−0.0065	0.2666	0.2780	0.957	0.2667	1.1043
	$\beta_{2,2}$	0.0047	0.1295	0.1301	0.939	0.1296	0.0060	0.1444	0.1416	0.930	0.1445	1.2434
60%	$\beta_{1,1}$	0.0076	0.3573	0.3758	0.953	0.3574	0.0128	0.4023	0.4189	0.955	0.4025	1.2678
	$\beta_{1,2}$	−0.0006	0.1626	0.1668	0.938	0.1626	−0.0035	0.2148	0.2053	0.926	0.2148	1.7451
	$\beta_{2,1}$	−0.0181	0.3520	0.3726	0.948	0.3525	−0.0318	0.3989	0.4179	0.949	0.4002	1.2842
	$\beta_{2,2}$	0.0037	0.1620	0.1645	0.939	0.1620	0.0154	0.2143	0.2036	0.918	0.2149	1.7499
80%	$\beta_{1,1}$	0.0339	0.5782	0.6052	0.956	0.5792	0.0431	1.4561	2.0539	0.979	1.4567	6.3420
	$\beta_{1,2}$	0.0195	0.2236	0.2244	0.930	0.2244	0.0057	0.3513	0.3565	0.932	0.3513	2.4684
	$\beta_{2,1}$	−0.0449	0.5725	0.5983	0.949	0.5743	−0.1274	1.5371	2.0190	0.961	1.5424	7.2086
	$\beta_{2,2}$	−0.0154	0.2197	0.2200	0.923	0.2202	0.0408	0.3515	0.3533	0.923	0.3539	2.5597

AIPW: augmented inverse probability weighting; Param: parameter; Bias: estimated bias; SD: standard deviation; ESE: estimated standard error; CP: coverage probability.

## 4. Application

Now we apply the proposed method to the motivating data from the ADNI study, a longitudinal study designed to develop clinical, imaging, genetic, and biochemical biomarkers for the early detection and tracking of AD. A pivotal advancement in the investigation of AD centers on the continuum spanning from the initial diagnosis to the progression of the disease and, ultimately, mortality. Meanwhile, the death of individuals with AD may be censored due to other causes, such as heart attack or pneumonia, resulting in a competing risk scenario. The true cause of death may also be missing due to either insufficient relevant information or delays in reporting follow-up data. Also, due to the nature of the study, the exact time of AD diagnosis is only known to fall between the last observed time before the conversion had not yet occurred and the first observed time when it had already taken place. In other words, the available data on survival time is restricted to interval-censored data.

In the following analysis, we focus on the participants in the ADNI 1 and 2 phases who recorded AD conversion during their follow-up visits. This results in a sample of 256 patients, among whom 21 participants experienced or reported death. Among the deceased individuals, one-third were confirmed to have died from AD progression, another one-third were from other causes, while the cause of death for the rest individuals could not be confirmed. We considered three covariates from the last visit before AD conversion: age (AGE), the APOE4 gene, and the AD assessment score (ADAS13). In addition to the proposed procedure with the mixture model, we applied three other methods: the proposed procedure with the Fine-Gray model, CC analysis with the mixture model, and the AIPW approach with the Fine-Gray model. The results are reported in Table 6 with the continuous covariates normalized. In the table, “others” refers to the cause of death rather than AD.

**Table 6.** Estimated covariate effects for the ADNI study.

		Mixture model			Fine-Gray model		
Index	Factors	Est	Se	p -val	Est	Se	p -val
		Proposed method			Proposed method		
AD	AGE	−1.0809	0.7440	0.1463	0.1213	0.3889	0.7551
	APOE4	−0.8105	2.6812	0.7624	0.1097	0.9672	0.9097
	ADAS13	−2.0279	0.7743	0.0088	−0.4200	0.4702	0.3717
Others	AGE	0.6963	0.4797	0.1466	0.3642	0.3485	0.2960
	APOE4	−0.4012	1.1629	0.7301	−0.2793	0.7951	0.7253
	ADAS13	0.6979	0.7590	0.3578	0.1515	0.2786	0.5866
Logistics	CONSTANT	−1.0880	1.6877	0.5191	–	–	–
	AGE	0.7833	0.5641	0.1650	–	–	–
	APOE4	0.0899	1.7521	0.9591	–	–	–
	ADAS13	1.1705	0.7705	0.1287	–	–	–
		Complete case analysis			AIPW		
AD	AGE	−1.4627	0.7583	0.0538	0.2425	0.3867	0.5307
	APOE4	−6.3738	2.0073	0.0015	0.0460	0.8307	0.9558
	ADAS13	−0.0156	0.5582	0.9777	−0.3803	0.4729	0.4213
Others	AGE	0.5294	0.4539	0.2434	0.2727	0.3529	0.4397
	APOE4	0.5735	0.9440	0.5435	−0.2676	0.8032	0.7390
	ADAS13	0.1335	0.4031	0.7404	0.1467	0.2858	0.6079
Logistics	CONSTANT	−4.5015	1.0649	0.0000	–	–	–
	AGE	0.8099	0.5727	0.1573	–	–	–
	APOE4	3.1343	1.6198	0.0530	–	–	–
	ADAS13	−0.8475	0.6973	0.2242	–	–	–

“AD” refers to death due to AD progression; “Others” refers to death from other causes; “Logistics” refers to the multinomial logistic regression component. ADNI: Alzheimer’s disease neuroimaging initiative; AD: Alzheimer’s disease.

Under the mixture model, the proposed method identifies ADAS13 as a significant predictor of AD progression, which aligns with the conclusion from existing literature.^47^ In contrast, neither of the two Fine-Gray model-based approaches nor the CC analysis identified any significant covariate effects on the CIF for AD progression. Particularly, the Fine-Gray model did not identify any significant predictors likely due to the different quantities described by these models. While the proposed mixture model separates a covariate’s effect on event probability from its effect on conditional event timing, the Fine-Gray model estimates the overall impact on the cumulative incidence. The significant effect of the covariate on the timing of AD-related death can therefore be diluted by competing risks, leading to a non-significant finding in the Fine-Gray analysis. The CC analysis instead identified APOE4 as significant, which may be due to the potential bias from excluding subjects with missing event types. Note that some regression parameter estimates have larger SEs under the proposed method, compared to the CC analysis. This slight discrepancy from our simulation results may be due to the missingness mechanism associated with the AD data being more complex than the MAR assumption we made. As shown in Table 2, however, the CC estimates often come at the cost of increased bias especially in the presence of a high MR, potentially leading to misleading conclusions about the significance of covariate effects. Similar observations were also reported by Guo et al.^32^ when the MAR assumption is violated.

**Figure 3.**
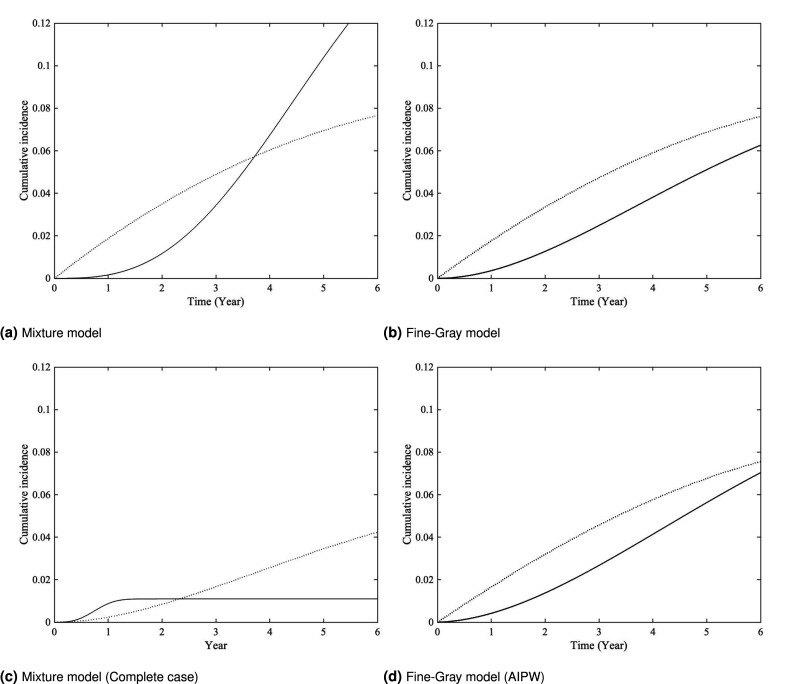
Estimated baseline CIF for the AD patients who died from AD (solid) versus other causes (dotted). Plots (a) and (b) are based on the proposed estimation method, while plots (c) and (d) are based on the complete-case analysis and two-stage AIPW, respectively. CIF: cumulative incidence function; AD: Alzheimer’s disease; AIPW: augmented inverse probability weighted.

**Figure 4.**
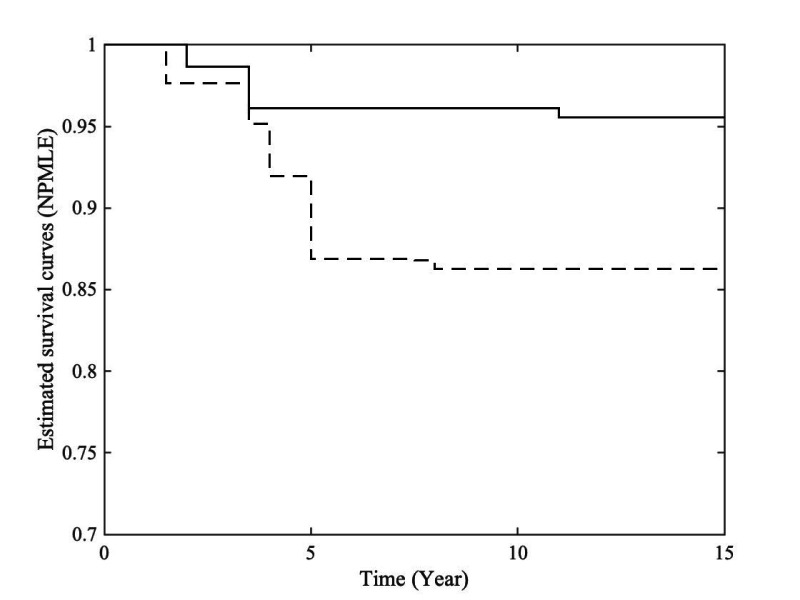
Estimated survival curves for the patients with covariate ADAS13: lower (dash-dot) or higher (solid) than the mean value.

Figure 3 presents the estimated baseline CIFs, defined as the estimated CIFs when all covariates are set to pseudo-values of zero. More specifically,

$${\hat{F}}_{k,0}\;(t)={\hat{\pi }}_{k}\;[1-\exp\;\{-{\hat{\Lambda }}_{k}(t)\}]\;,\;k=1,\;2$$

for the mixture model, and

$${\hat{F}}_{k,0}^{\;FG}\;(t)=1-\exp\;\{-{\hat{\Lambda }}_{k}^{\;FG}(t)\},\;k=1,\;2$$

for the Fine-Gray model. From the figure, we observe that the estimated curves under the mixture model exhibit a crossover around the 4-year mark in plot (a). The difference in crossover timing and curve behavior between plots (a) and (c) may be attributed to the exclusion of cases with missing causes of death in the latter. However, both methods based on the Fine-Gray model fail to capture this crossing pattern in plots (b) and (d), possibly indicating some limitations of the Fine-Gray model for this specific application. Figure 4 presents the estimated conditional survival functions for individuals who died from AD, stratified by whether their ADAS13 values were above or below the mean, obtained using Turnbull’s NPMLE to account for interval censoring. The graphical results further support the significant effect of ADAS13 on the progression from AD to death.

## 5. Conclusions

In this article, we discussed regression analysis of interval-censored competing risk data with missing event types and for the problem, a novel direct likelihood mixture mode approach was proposed. One advantage of the proposed approach is that it avoids the modeling of the missing type indicator, which is often at risk of being misspecified due to its unobservable nature. Also the proposed estimation procedure is more computationally appealing and results in more efficient estimates than the existing method.

As noted above, the approach for handling missing event types can be applied to other competing risks models, such as the cause-specific hazard model and the subdistribution hazard model, including the Fine-Gray model. However, to the best of our knowledge, no existing test procedure assesses model assumptions under interval-censored competing risks data, which warrants further investigation. While we use a mixture distribution model, which offers a different and computationally simpler structure compared to the conventional cause-specific or subdistribution hazard model, its performance depends on the availability of sufficient observations for each event type. A larger number of observed events provide more information for the logistic models, enhancing estimation accuracy.

Another important consideration is that different models yield different interpretations of covariate effects.^48^ For instance, the effect of covariates on the cause-specific hazard may differ substantially from their effect on the subdistribution hazard and can even be in the opposite direction./^12,49^ By definition, subdistribution hazard-based approaches measure covariate effects that directly influence the CIF, whereas cause-specific hazard approaches capture covariate effects that are not directly related to the CIF. Thus, the choice of model should align with the specific research objective, the desired interpretation of covariate effects, and the availability of data.

Finally, there exist some important directions for future research. One is that in the preceding sections, we have focused on time-independent covariates. Generalizing the proposed framework to handle time-dependent covariates is a crucial next step although this introduces significant computational challenges due to the integrals involved in modeling the covariate history.^50,51^ Another possible extension concerns the assumption of non-informative censoring. When the observation process is dependent on the failure time or one faces informative interval censoring, a joint modeling approach for both the failure and censoring mechanisms would be necessary.^52^ Developing these extensions presents a valuable avenue for future investigation.

## Supplemental Material

sj-pdf-1-smm-10.1177_09622802261420820 - Supplemental material for Regression analysis of interval-censored competing risks data with missing causes of failure: A direct likelihood approach Supplemental material, sj-pdf-1-smm-10.1177_09622802261420820 for Regression analysis of interval-censored competing risks data with missing causes of failure: A direct likelihood approach by Yichen Lou, Yuqing Ma, Liming Xiang and Jianguo Sun in Statistical Methods in Medical Research

## Appendix: Generalization to Fine-Gray model and cause-specific hazard model

In this appendix, we provide a detailed explanation of Remark 2 in Section 2.2, discussing how the idea of the proposed method extends to the Fine-Gray model and the cause-specific hazard model. Specifically, we consider cases where the primary interest lies in estimating the regression parameters β in the CIF or the CIF itself.

For the Fine-Gray model, the CIF is given by the following equation:

$$F_{k}^{\;FG}\;(t;\;Z)=1-\exp\;\{-\Lambda_{k}^{\;FG}(t)\;\exp\;(\beta_{k}^{⊤}\;Z)\}$$

for cause k , where $\Lambda_{k}^{\;FG}$ is an unspecified strictly increasing function, and k=1,…,K . The likelihood function is

$$\begin{array}{rl} \ell_{FG}(\theta ) & =[\{F_{k}^{\;FG}(L_{i};\;Z){\}}^{{\tilde{\Delta }}_{ik}}\{F_{k}^{\;FG}(R_{i};\;Z)\;-\;F_{k}^{\;FG}(L_{i};\;Z){\}}^{{\bar{\Delta }}_{ik}}{]}^{\Delta_{i}\chi_{i}}\\ & \;\times [\{1-S^{\;FG}(L_{i};\;Z){\}}^{{\tilde{\Delta }}_{i}}\{S^{\;FG}(L_{i};\;Z)-S^{\;FG}(R_{i};\;Z){\}}^{{\bar{\Delta }}_{i}}{]}^{\Delta_{i}(1-\chi_{i})}\{S^{\;FG}(R_{i};\;Z){\}}^{1-\Delta_{i}} \end{array}$$

where

$$S^{\;FG}\;(t;\;Z)=1-\sum_{k=1}^{K}F_{k}^{\;FG}\;(t;\;Z)$$

is the overall survival function. To ensure identifiability, the likelihood function $\ell_{FG}(\theta )$ must satisfy the constraint

$$\max_{Z}\left\{\sum_{k=1}^{K}F_{k}^{\;FG}\;(t;\;Z)\right\}<1$$

For optimization, following Lou et al.,^10^ we approximate $\Lambda_{k}^{\;FG}$ using the same sieve method described in Section 2.2 before optimization.

For the cause-specific hazard (CSH) model, the CIF is given by the following equation:

$$F_{k}^{\;CSH}\;(t;\;Z)=\int_{0}^{⊤}\;\lambda_{k}^{\;CSH}(s)\;\exp\;(Z^{⊤}\beta_{k})\;\exp\;\{-\int_{0}^{s}\sum_{j=1}^{K}\;\lambda_{j}^{\;CSH}(v)\;\exp\;(Z^{⊤}\beta_{j})\;dv\}\;ds\;$$

for cause k , where k=1,…,K . The corresponding likelihood function is

$$\begin{array}{rl} \ell_{CSH}(\theta ) & =[\{F_{k}^{\;CSH}(L_{i}\;Z){\}}^{{\tilde{\Delta }}_{ik}}\{F_{k}^{\;CSH}(R_{i};\;Z)\;-\;F_{k}^{\;CSH}(L_{i};\;Z){\}}^{{\bar{\Delta }}_{ik}}{]}^{\Delta_{i}\chi_{i}}\\ & \;\times [\{1-S^{\;CSH}(L_{i};\;Z){\}}^{{\tilde{\Delta }}_{i}}\{S^{\;CSH}(L_{i};\;Z)-S^{\;CSH}(R_{i};\;Z){\}}^{{\bar{\Delta }}_{i}}{]}^{\Delta_{i}(1-\chi_{i})}\{S^{\;CSH}(R_{i};\;Z){\}}^{1-\Delta_{i}} \end{array}$$

where

$$S^{\;CSH}\;(t;\;Z)=1-\sum_{k=1}^{K}F_{k}^{\;CSH}\;(t;\;Z)$$

Estimating $\ell_{CSH}(\theta )$ is more challenging due to the involvement of both unknown functions and integrals without analytical solutions. To address this, we first approximate $\Lambda_{k}$ using the sieve method described in Section 2.2, and evaluate the integral via Gauss-Jacobi quadrature. The number of quadrature points is chosen proportional to the interval length, typically set to five, as this has been shown to provide sufficient accuracy for numerical integration.^8^

Therefore, the parameters in the CIF or the CIF itself can be estimated using different competing risk models.
