# Supplementary material for: Regression analysis of interval-censored competing risks data with missing causes of failure: A direct likelihood approach
Source: Stat Methods Med Res. 2026 Mar 4;35(4):795–811. doi: 10.1177/09622802261420820 (PMC13161494; doi:10.1177/09622802261420820)
Supplement: sj-pdf-1-smm-10.1177_09622802261420820 - Supplemental material for Regression analysis of interval-censored competing risks data with missing causes of failure: A direct likelihood approach [file sj-pdf-1-smm-10.1177_09622802261420820.pdf]

Supplemental Material for " Regression Analysis of Interval-Censored Competing Risks Data with Missing Causes of Failure: A Direct Likelihood Approach "

Yichen Lou, Yuqing Ma, Liming Xiang*, Jianguo Sun

1 Proofs of the Asymptotic Properties

In this appendix, we will sketch the proofs of the asymptotic properties given in Section 3. Empirical process theory techniques will be applied to derive the consistency of the proposed sieve maximum likelihood estimator. Following the notation used in van der Vaart and Wellner (1996) [11], throughout the appendix, we denote $\mathcal{P}f = \int_{\mathcal{X}} f(x)\, d\,\mathbb{P}(x)$ and $\mathbb{P}_n f = n^{-1}\sum_{i=1}^{n} f(X_i)$, which is the empirical process indexed by the function f evaluated as X_i, the sample points of individual i, with $\mathcal{X}$ denoting the sample space. To avoid confusion, we add a subscript n to all the estimators in the following, for example, the estimators $\widehat{\boldsymbol{\beta}}_n$, $\widehat{\boldsymbol{\theta}}_n$ and $\widehat{\Lambda}_{kn}$. Let $\ell(\boldsymbol{\theta}; O)$ denote the log-likelihood function based on a single observation O, and the letter C represents a constant, which does not necessarily represent the same value each time here and in the proof. To begin with, we will first describe the regularity conditions needed.

Condition 1. *The failure time and event of interest* (T, D) *and observation process* $\boldsymbol{V}$ *are independent given the covariate* $(\boldsymbol{Z}, \boldsymbol{W})$*. Furthermore, there exists a positive number* $\xi > 0$ *such that* $\mathbb{P}(V_{j+1} - V_j \geq \xi \mid \boldsymbol{Z}, \boldsymbol{W}) = 1$*, and the number of examination times* J *is positive with* $\mathbb{E}(J) < \infty$*.*

Condition 2. *(a)* $\boldsymbol{Z}$ *belongs to a bounded subset of* $\mathbb{R}^p$*; (b)* $\boldsymbol{W}$ *belongs to a bounded subset of* $\mathbb{R}^q$*.*

Condition 3. *(a)* $\boldsymbol{\eta}_0 = \{\boldsymbol{\beta}_{k0}, \boldsymbol{\gamma}_0; k = 1, \dots K\} \in \mathcal{B}$ *and* $\mathcal{B}$ *is compact subset of* $\mathbb{R}^{Kp+(K-1)(q+1)}$*; (b) The function* $\Lambda_{k0}(\cdot)$ *is continuously differentiable of at least order* r *in* $[a, b]$ *and satisfies* $\xi^{-1} < \Lambda_{k0}(a) < \Lambda_{k0}(b) < \xi$ *for some positive constant* ξ*,* $k = 1, \dots, K$*; (c) If there exist* $A(t)$ *and* $\mathbf{c}$ *such that* $A(t) + \mathbf{c}^\top \boldsymbol{Z} = 0$ *with probability one, then* $A(t) = 0$ *and* $\mathbf{c} = \mathbf{0}$*.*

Condition 4. *For every* $\boldsymbol{\theta}$ *in a neighborhood of* $\boldsymbol{\theta}_0$*, we have that* $\mathcal{P}\ell(\boldsymbol{\theta}, O) - \mathcal{P}\ell(\boldsymbol{\theta}_0, O) \preceq -d^2(\boldsymbol{\theta}, \boldsymbol{\theta}_0)$*, where* $\mathcal{P}$ *denotes the measure defined above and* $\preceq$ *means 'smaller than, up to a constant'.*

Note that Condition 1 specifies an independent censoring scheme [6], while Conditions 2 – 4 are commonly used in the analysis of interval-censored failure time data [3, 12]. In particular, Condition 3 ensures the smoothness of the sieve approximation.

Proof of consistency. The consistency of the proposed estimator $\widehat{\boldsymbol{\theta}}_n$ can be established by using Theorem 5.7 of van der Vaart (2000) [10]. To this end, we need to check the following three conditions:

(i) $\sup_{\boldsymbol{\theta}\in\Theta_n}|M_n(\boldsymbol{\theta})-M(\boldsymbol{\theta})|\to 0$;

(ii) $\sup_{\boldsymbol{\theta}:d(\boldsymbol{\theta},\boldsymbol{\theta}_0)>\epsilon}M(\boldsymbol{\theta})<M(\boldsymbol{\theta}_0)$;

(iii) $M_n(\widehat{\boldsymbol{\theta}}_n)\geq M_n(\boldsymbol{\theta}_0)-o_p(1)$;

for $M(\boldsymbol{\theta})=\mathcal{P}\ell(\boldsymbol{\theta};O)$ and $M_n(\boldsymbol{\theta})=\mathbb{P}_n\ell(\boldsymbol{\theta};O)$. For condition (i), consider the class of functions $\mathcal{F}_n=\{\ell(\boldsymbol{\theta};O):\boldsymbol{\theta}\in\Theta_n\}$. Then, by Corollary 3.2 in Arcones and Gine (1993) [1], we only need to prove the covering number of $\mathcal{F}_n$ satisfies $\log N(\epsilon,\mathcal{F}_n,L_1(\mathbb{P}_n))/n\to 0$. First note that for any $\boldsymbol{\theta}^{(1)}=(\boldsymbol{\eta}^{(1)},\Lambda_k^{(1)};k=1,\ldots,K)\in\Theta_n$ and $\boldsymbol{\theta}^{(2)}=(\boldsymbol{\eta}^{(2)},\Lambda_k^{(2)};k=1,\ldots,K)\in\Theta_n$, one can easily obtain that

$$|\ell(\boldsymbol{\theta}^{(1)};O)-\ell(\boldsymbol{\theta}^{(2)};O)|\leq C\Big\{||\boldsymbol{\eta}^{(1)}-\boldsymbol{\eta}^{(2)}||+\sum_{k=1}^{K}||\Lambda_k^{(1)}-\Lambda_k^{(2)}||_\infty\Big\}$$

by using Taylor's series expansion under Conditions 1, 2 and 3. Denote $\boldsymbol{\psi}_k^{(1)}=\{\psi_k^{0(1)},\ldots,\psi_k^{m(1)}\}$ and $\boldsymbol{\psi}_k^{(2)}=\{\psi_k^{0(2)},\ldots,\psi_k^{m(2)}\}$ the Bernstein coefficients corresponding to $\Lambda_k^{(1)}$ and $\Lambda_k^{(2)}$, respectively, $k=1,\ldots,K$. Then it is easy to show that

$$\begin{aligned}||\Lambda_k^{(1)}-\Lambda_k^{(2)}||_\infty&=\sup_t\Big|\sum_{j=0}^{m}\psi_k^{j(1)}B_j(t,m,a,b)-\sum_{j=0}^{m}\psi_k^{j(2)}B_j(t,m,a,b)\Big|\\&\leq\max_{0\leq j\leq m}|\psi_k^{j(1)}-\psi_k^{j(2)}|:=||\boldsymbol{\psi}_k^{(1)}-\boldsymbol{\psi}_k^{(2)}||\,.\end{aligned}$$

Thus, we obtain

$$|\ell(\boldsymbol{\theta}^{(1)};O)-\ell(\boldsymbol{\theta}^{(2)};O)|\leq C\Big\{||\boldsymbol{\eta}^{(1)}-\boldsymbol{\eta}^{(2)}||+\sum_{k=1}^{K}||\boldsymbol{\psi}_k^{(1)}-\boldsymbol{\psi}_k^{(2)}||\Big\}\,.$$

By Lemma 2.5 of [9], for $\{\boldsymbol{\eta}\in\mathbb{R}^{Kp+(K-1)(q+1)},\ ||\boldsymbol{\eta}||\leq M\}$ and $\{\boldsymbol{\psi}_k\in\mathbb{R}^{m+1},\ \sum_{0\leq j\leq m}|\psi^j|\leq M_n\}$, one can show that the former is covered by $[5M/\{\epsilon/(4C)\}]^{Kp+(K-1)(q+1)}$ balls with radius $\epsilon/(4C)$, and the latter is covered by $[5M_n/\{\epsilon/(4C)\}]^{m+1}$ balls with radius $\epsilon/(4C)$. Thus we have

$$\begin{aligned}N(\epsilon,\mathcal{F}_n,L_1(\mathbb{P}_n))&\leq C\left(\frac{20CM}{\epsilon}\right)^{Kp+(K-1)(q+1)}\left(\frac{20CM_n}{\epsilon}\right)^{K(m+1)}\\&\leq C{M_n}^{(m+1)}\epsilon^{-[Kp+(K-1)(q+1)+K(m+1)]}\,.\end{aligned}$$

In this way, one can show that $\log N(\epsilon,\mathcal{F}_n,L_1(\mathbb{P}_n))/n\to 0$. Thus, we have $\sup_{\theta\in\Theta_n}|M_n(\boldsymbol{\theta})-M(\boldsymbol{\theta})|\to 0$.

For condition (ii), first note that the Gibbs inequality implies that $\sup_{\boldsymbol{\theta}:d(\boldsymbol{\theta},\boldsymbol{\theta}_0)>\epsilon}M(\boldsymbol{\theta})\leq M(\boldsymbol{\theta}_0)$. Assume that $\sup_{\boldsymbol{\theta}:d(\boldsymbol{\theta},\boldsymbol{\theta}_0)>\epsilon}M(\boldsymbol{\theta})=M(\boldsymbol{\theta}_0)$. Then there exists a sequence $\boldsymbol{\theta}_m$ such that $M(\boldsymbol{\theta}_m)\to\sup_{\boldsymbol{\theta}:d(\boldsymbol{\theta},\boldsymbol{\theta}_0)>\epsilon}M(\boldsymbol{\theta})$ and $d(\boldsymbol{\theta}_m,\boldsymbol{\theta}_0)>\epsilon$. By Condition 3, $\mathcal{B}$ is compact, and the sieve coefficients are bounded, there exists a subsequence $\boldsymbol{\theta}_{\widetilde{m}}$ converging to $\boldsymbol{\theta}_{m0}$. Since $M(\boldsymbol{\theta})$ is a continuous function of $\boldsymbol{\theta}$, $M(\boldsymbol{\theta}_{m0})=M(\boldsymbol{\theta}_0)$ and consequently $\boldsymbol{\theta}_{m0}=\boldsymbol{\theta}_0$ according to the identifiability of the proposed

model. However, $\boldsymbol{\theta}_{\widetilde{m}}$ does not converge to $\boldsymbol{\theta}_0$ due to the fact $d(\boldsymbol{\theta}_{\widetilde{m}}, \boldsymbol{\theta}_0) > \epsilon$. This conflicts with the aforementioned results that $\boldsymbol{\theta}_{\widetilde{m}}$ converges to $\boldsymbol{\theta}_{m0}$. Therefore, we have that $\sup_{\boldsymbol{\theta}: d(\boldsymbol{\theta}, \boldsymbol{\theta}_0) > \epsilon} M(\boldsymbol{\theta}) < M(\boldsymbol{\theta}_0)$.

For condition (iii), define $\boldsymbol{\theta}_{0,n} = (\boldsymbol{\eta}_0, \Lambda_{kn};\ k = 1, \ldots, K)$. We first have

$$\begin{aligned} M_n(\widehat{\boldsymbol{\theta}}_n) - M_n(\boldsymbol{\theta}_0) &= M_n(\widehat{\boldsymbol{\theta}}_n) - M_n(\boldsymbol{\theta}_{0,n}) + M_n(\boldsymbol{\theta}_{0,n}) - M_n(\boldsymbol{\theta}_0) \\ &\geq \mathbb{P}_n \ell(\boldsymbol{\theta}_{0,n}; O) - \mathbb{P}_n \ell(\boldsymbol{\theta}_0; O) \\ &= (\mathbb{P}_n - \mathcal{P})\Big\{\ell(\boldsymbol{\theta}_{0,n}; O) - \ell(\boldsymbol{\theta}_0; O)\Big\} - \mathcal{P}\Big\{\ell(\boldsymbol{\theta}_0; O) - \ell(\boldsymbol{\theta}_{0,n}; O)\Big\}. \end{aligned}$$

First, note from Theorem 1.6.2 of Lorentz (1986) [5] that there exists a Bernstein polynomial Λ_{kn} such that $||\Lambda_{kn} - \Lambda_{k0}||_\infty = O(m^{-r/2})$, $k = 1, \ldots, K$. Then we have $d(\boldsymbol{\theta}_{0,n}, \boldsymbol{\theta}_0) = O(n^{-r\nu/2})$. Thus, by following Taylor's expansion, one can show that $\mathcal{P}\{\ell(\boldsymbol{\theta}_0; O) - \ell(\boldsymbol{\theta}_{0,n}; O)\} \leq C d^2(\boldsymbol{\theta}_{0,n}, \boldsymbol{\theta}_0)$. As $n \to \infty$, we have $\mathcal{P}\{\ell(\boldsymbol{\theta}_0; O) - \ell(\boldsymbol{\theta}_{0,n}; O)\} \to 0$, thus $\mathcal{P}\{\ell(\boldsymbol{\theta}_0; O) - \ell(\boldsymbol{\theta}_{0,n}; O)\} = o(1)$. Secondly, we show that $(\mathbb{P}_n - \mathcal{P})\{\ell(\boldsymbol{\theta}_{0,n}; O) - \ell(\boldsymbol{\theta}_0; O)\} = o_p(n^{-1/2})$. Define $\mathcal{F}_{\widetilde{n}} = \{\ell(\boldsymbol{\theta}; O) - \ell(\boldsymbol{\theta}_{0,\widetilde{n}}; O) : \boldsymbol{\theta} \in \Theta_n, ||\boldsymbol{\theta}_0 - \boldsymbol{\theta}_{0,\widetilde{n}}|| \leq Cn^{-r\nu/2}\}$. Clearly, $\ell(\boldsymbol{\theta}_0; O) - \ell(\boldsymbol{\theta}_{0,n}; O) \in \mathcal{F}_{\widetilde{n}}$. Similar to the proof of the bounded bracket number of $\mathcal{F}_n$, we can prove that the ϵ bracketing number is also bounded by $C(1/\epsilon)^{[K(m+1)]}$. Moreover, we have that

$$J_{[]}(\delta, \mathcal{F}_{\widetilde{n}}, L_2(\mathcal{P})) = \int_0^\delta \sqrt{\log N(\epsilon, \mathcal{F}_{\widetilde{n}}, L_2(\mathcal{P}))} d\epsilon \leq \int_0^\delta \sqrt{C[K(m+1)] \log 1/\epsilon} < \infty$$

by the bracketing integral defined in pp.270 of van der Vaart (2000) [10]. Thus, $F_{\widetilde{n}}$ is a P-Donsker by Theorem 19.5 in van der Vaart (2000) [10]. According to Corollary 2.3.12 in van der Vaart and Wellner (1996) [11], we have $(\mathbb{P}_n - \mathcal{P})\{\ell(\boldsymbol{\theta}_{0,n}; O) - \ell(\boldsymbol{\theta}_0; O)\} = o_p(n^{-1/2})$. Thus, we obtain that $M_n(\widehat{\boldsymbol{\theta}}_n) - M_n(\boldsymbol{\theta}_0) \geq o_p(n^{-1/2}) - o(1) = -o_p(1)$.

Now, three conditions of Theorem 5.7 of van der Vaart (2000) [10] are verified, and we can complete the proof and obtain that $d(\widehat{\boldsymbol{\theta}}_n, \boldsymbol{\theta}_0) \to 0$ in probability.

□

Proof of rate of convergence. We prove this theorem by applying Theorem 3.4.1 of van der Vaart and Wellner (1996) [11] in which two conditions have to be satisfied.

The first condition is that for every n and an arbitrary δ with $\delta > \delta_n = n^{-r\nu/2}$,

$$\sup_{\delta/2 < d(\boldsymbol{\theta}, \boldsymbol{\theta}_{0,n}) < \delta, \theta \in \Theta} \Big\{M(\boldsymbol{\theta}) - M(\boldsymbol{\theta}_{0,n})\Big\} \leq -C\delta^2.$$

From the proof of the consistency, under Conditions 1-3, we have $d(\boldsymbol{\theta}_0, \boldsymbol{\theta}_{0,n}) = O(n^{-r\nu/2})$ and $M(\boldsymbol{\theta}_0) - M(\boldsymbol{\theta}_{0,n}) \leq C d^2(\boldsymbol{\theta}_{0,n}, \boldsymbol{\theta}_0) \leq O(n^{-r\nu})$. Moreover, under Condition 4, we have

$$M(\boldsymbol{\theta}) - M(\boldsymbol{\theta}_0) = \mathcal{P}\ell(\boldsymbol{\theta}; O) - \mathcal{P}\ell(\boldsymbol{\theta}_0; O) \leq -C\, d^2(\boldsymbol{\theta}, \boldsymbol{\theta}_0) \leq -C\delta^2$$

for every $\boldsymbol{\theta}$ in the neighbourhood of $\boldsymbol{\theta}_0$. Then, for a large enough n, we have

$$M(\boldsymbol{\theta}) - M(\boldsymbol{\theta}_{0,n}) = M(\boldsymbol{\theta}) - M(\boldsymbol{\theta}_0) + M(\boldsymbol{\theta}_0) - M(\boldsymbol{\theta}_{0,n}) \le -C\delta^2 + Cn^{-r\nu} \le -C\delta^2 \,,$$

and so the first condition in Theorem 3.4.1 of van der Vaart and Wellner (1996) [11] is satisfied.

The second condition to be verified is that there exists a proper function $\phi(\cdot)$ such that

$$\mathbb{E}\Big\{ \sup_{\delta/2 < d(\boldsymbol{\theta},\boldsymbol{\theta}_{0,n}) < \delta} (M_n - M)(\boldsymbol{\theta} - \boldsymbol{\theta}_{0,n}) \Big\}^+ \le C\frac{\phi(\delta)}{\sqrt{n}}$$

hold for small enough δ, where $\delta \to \phi(\delta)/\delta^{\xi}$, which is a decreasing function of δ for some $\xi < 2$, and there exists $r_n \le \delta_n^{-1}$ for every n such that $r_n^2\phi(1/r_n) \le C\sqrt{n}$. To see this, define a class $\mathcal{F}_\delta = \{\ell(\boldsymbol{\theta}; O) - \ell(\boldsymbol{\theta}_{0,n}; O) : \boldsymbol{\theta} \in \Theta_n, \delta/2 < d(\boldsymbol{\theta}, \boldsymbol{\theta}_{0,n}) < \delta\}$. One can easily show that $\mathcal{P}\{\ell(\boldsymbol{\theta}_0; O) - \ell(\boldsymbol{\theta}_{0,n}; O)\} \le Cd^2(\boldsymbol{\theta}_{0,n}, \boldsymbol{\theta}_0) \le Cn^{-r\nu}$. Hence, under Condition 4 and similar arguments before, we have for large n, $\mathcal{P}\{\ell(\boldsymbol{\theta}_0; O) - \ell(\boldsymbol{\theta}_{0,n}; O)\} \le C\delta^2$. By similar arguments in the proof of the consistency and Lemma 5 in Shen and Wong (1994) [8], we have that $\log N_{[]}(\epsilon, \mathcal{F}_\delta, L_2(\mathcal{P})) \le CN\log(\delta/\epsilon)$ with $N = K(m+1)$. Moreover, we have

$$J_{[]}(\delta, \mathcal{F}_\delta, L_2(\mathcal{P})) = \int_0^\delta \sqrt{1 + \log N_{[]}(\epsilon, \mathcal{F}_\delta, L_2(\mathcal{P}))}\, d\epsilon \le CN^{1/2}\delta \,.$$

Therefore, under Conditions 1 – 3, Lemma 3.4.2 of van der Vaart and Wellner (1996) [11] and similar arguments in Zhou et al. (2017) [12], it is easy to see that $\mathcal{F}_\delta$ is uniformly bounded, and therefore

$$\mathbb{E}_{\mathcal{P}}\Big\{\sqrt{n}||\mathbb{P}_n - \mathcal{P}||_{\mathcal{F}_\delta}\Big\} \le CJ_{[]}(\delta, \mathcal{F}_\delta, L_2(\mathcal{P}))\Big(1 + \frac{J_{[]}(\delta, \mathcal{F}_\delta, L_2(\mathcal{P}))}{\delta^2 n^{1/2}}\Big) \,.$$

Thus, we can choose $\phi(\delta) = N^{1/2}\delta + Nn^{-1/2}$. The condition that $\phi(\delta)/\delta$ is a decreasing function of δ is satisfied, and if we choose r_n to be $N^{-1/2}n^{1/2} = n^{(1-\nu)/2}$, then $r_n^2\phi(1/r_n) \le Cn^{1/2}$ is satisfied. The condition $M_n(\widehat{\boldsymbol{\theta}}_n) \ge M_n(\boldsymbol{\theta}_{0,n})$ is clearly satisfied, as $\widehat{\boldsymbol{\theta}}_n$ maximizes the likelihood. Finally, $d(\widehat{\boldsymbol{\theta}}_n, \boldsymbol{\theta}_{0,n}) \le d(\widehat{\boldsymbol{\theta}}_n, \boldsymbol{\theta}_0) + d(\boldsymbol{\theta}_0, \boldsymbol{\theta}_{0,n}) \to 0$ in probability. By Theomre 3.4.1 of van der Vaart and Wellner (1996) [11], we get that $r_n d(\widehat{\boldsymbol{\theta}}_n, \boldsymbol{\theta}_{0,n}) = O_p(1)$. Together with the fact that $d(\boldsymbol{\theta}_0, \boldsymbol{\theta}_{0,n}) = O(n^{-r\nu/2})$, we obatin that

$$d(\widehat{\boldsymbol{\theta}}_n, \boldsymbol{\theta}_0) = O_p\Big(n^{-(1-\nu)/2} + n^{-r\nu/2}\Big) \,.$$

□

Proof of asymptotic normality and efficiency. Let Υ denote a linear span of $\Theta - \boldsymbol{\theta}_0$, where $\boldsymbol{\theta}_0$ is the true value, and Θ is the original parameter space. Define the first order directional derivative of $\ell(\boldsymbol{\theta}; O)$ at the direction $\boldsymbol{\iota} \in \Upsilon$ and the second order directional derivative as

$$\ell'(\boldsymbol{\theta}; O)[\boldsymbol{\iota}] = \frac{\partial \ell(\boldsymbol{\theta} + s\boldsymbol{\iota}; O)}{\partial s}\Big|_{s=0}, \quad \ell''(\boldsymbol{\theta}; O)[\boldsymbol{\iota}, \widetilde{\boldsymbol{\iota}}] = \frac{\partial \ell(\boldsymbol{\theta} + s\boldsymbol{\iota} + \widetilde{s}\widetilde{\boldsymbol{\iota}}; O)}{\partial \widetilde{s}\partial s}\Big|_{s=0}\Big|_{\widetilde{s}=0}$$

for any $\boldsymbol{\theta} \in \{\boldsymbol{\theta} \in \Theta : d(\boldsymbol{\theta}, \boldsymbol{\theta}_0) = O(\delta_n)\}$, where $\delta_n = n^{-\min\{(1-\nu)/2, r\nu/2\}}$. Also let $\ell'(\boldsymbol{\theta}_0; O)[\boldsymbol{\iota}]$ and $\ell''(\boldsymbol{\theta}_0; O)[\boldsymbol{\iota}, \widetilde{\boldsymbol{\iota}}]$ denote $\ell'(\boldsymbol{\theta}; O)[\boldsymbol{\iota}]$ and $\ell''(\boldsymbol{\theta}; O)[\boldsymbol{\iota}, \widetilde{\boldsymbol{\iota}}]$ evaluated at $\boldsymbol{\theta}_0$, respectively. Define Fisher inner product on the space Υ as $< \boldsymbol{\iota}, \widetilde{\boldsymbol{\iota}} >= \mathcal{P}\{\ell'(\boldsymbol{\theta}_0; O)[\boldsymbol{\iota}]\ell'(\boldsymbol{\theta}_0; O)[\widetilde{\boldsymbol{\iota}}]\}$ and Fisher norm $||\boldsymbol{\iota}|| =< \boldsymbol{\iota}, \boldsymbol{\iota} >$. Let $\overline{\Upsilon}$ be the closed linear span of Υ under the Fisher norm. Then $(\overline{\Upsilon}, ||\cdot||)$ is a Hilbert space. For a vector of $(Kp + q + 1)$ dimension $\boldsymbol{\alpha} = (\boldsymbol{\alpha}_1^\top, \boldsymbol{\alpha}_2^\top)^\top$ with $\boldsymbol{\alpha}_1 = (\boldsymbol{\alpha}_{1,1}^\top, \ldots, \boldsymbol{\alpha}_{1,K}^\top)^\top$ and $||\boldsymbol{\alpha}|| \leq 1$ and for any $\boldsymbol{\iota} = (\boldsymbol{\iota}_\eta, \boldsymbol{\iota}_{\Lambda_k}; k = 1, \ldots, K) \in \Upsilon$, define a smooth function of $\boldsymbol{\theta}$ as $h(\boldsymbol{\theta}) = \boldsymbol{\alpha}_1^\top \boldsymbol{\beta} + \boldsymbol{\alpha}_2^\top \boldsymbol{\gamma} = \boldsymbol{\alpha}^\top \boldsymbol{\eta}$, where $\boldsymbol{\beta} = (\boldsymbol{\beta}_1^\top, \ldots, \boldsymbol{\beta}_K^\top)^\top$, and

$$h'(\boldsymbol{\theta}_0)[\boldsymbol{\iota}] = \frac{\partial h(\boldsymbol{\theta}_0 + s\boldsymbol{\iota})}{\partial s}\Big|_{s=0}$$

where the right-hand side limit is well-defined.

The proof of the asymptotic normality can be verified with the conditions and the Riesz representation theorem along the same line as that of Theorem 3 in Zhou et al. (2017) [12]. We first prove that there exists an $\boldsymbol{\iota}^* \in \Upsilon$ s.t.

$$n^{1/2} < \widehat{\boldsymbol{\theta}}_n - \boldsymbol{\theta}_0, \, \boldsymbol{\iota}^* > \Rightarrow \mathcal{N}\left(\mathbf{0}\,,\, \boldsymbol{\alpha}^\top I_*^{-1}(\boldsymbol{\eta}_0)\, \boldsymbol{\alpha}\right)$$

by first proving $n^{1/2} < \widehat{\boldsymbol{\theta}}_n - \boldsymbol{\theta}_0, \, \boldsymbol{\iota}^* > \Rightarrow \mathcal{N}(0, ||\boldsymbol{\iota}^*||^2)$ and then $||\boldsymbol{\iota}^*||^2 = \boldsymbol{\alpha}^\top I_*^{-1}(\boldsymbol{\eta}_0)\boldsymbol{\alpha}$. First note that by Theorem 1.6.2 of Lorentz (1986) [5], there exists $\Pi_n \boldsymbol{\iota}^* \in \Theta_n - \boldsymbol{\theta}_0$ such that $||\Pi_n \boldsymbol{\iota}^* - \boldsymbol{\iota}^*|| = o(n^{-\nu})$. Also note that with $r > 2$ and $\nu > 1/(2r)$, we have $\delta_n ||\Pi_n \boldsymbol{\iota}^* - \boldsymbol{\iota}^*|| = o(n^{-1/2})$. Define $\rho(\boldsymbol{\theta} - \boldsymbol{\theta}_0) = \ell(\boldsymbol{\theta}; O) - \ell(\boldsymbol{\theta}_0; O) - \ell'(\boldsymbol{\theta}_0; O)[\boldsymbol{\theta} - \boldsymbol{\theta}_0]$, and let ϵ_n be any positive sequence satisfying $\epsilon_n = o(n^{-1/2})$. By the definition of $\widehat{\boldsymbol{\theta}}_n$ and $\mathcal{P}\ell'(\boldsymbol{\theta}_0; O)[\Pi_n \boldsymbol{\iota}^*]$, we have

$$\begin{aligned}
0 \leq & \mathbb{P}_n \Big\{ \ell(\widehat{\boldsymbol{\theta}}_n; O) - \ell(\widehat{\boldsymbol{\theta}}_n \pm \epsilon_n \Pi_n \boldsymbol{\iota}^*; O) \Big\} \\
= & (\mathbb{P}_n - \mathcal{P}) \Big\{ \ell(\widehat{\boldsymbol{\theta}}_n; O) - \ell(\widehat{\boldsymbol{\theta}}_n \pm \epsilon_n \Pi_n \boldsymbol{\iota}^*; O \Big\} + \mathcal{P} \Big\{ \ell(\widehat{\boldsymbol{\theta}}_n; O) - \ell(\widehat{\boldsymbol{\theta}}_n \pm \epsilon_n \Pi_n \boldsymbol{\iota}^*; O) \Big\} \\
= & \mp \epsilon_n \mathbb{P}_n \ell'(\boldsymbol{\theta}_0; O)[\boldsymbol{\iota}^*] \mp \epsilon_n \mathbb{P}_n \ell'(\boldsymbol{\theta}_0; O)[\Pi_n \boldsymbol{\iota}^* - \boldsymbol{\iota}^*] + (\mathbb{P}_n - \mathcal{P}) \Big\{ \rho(\widehat{\boldsymbol{\theta}}_n - \boldsymbol{\theta}_0; O) - \rho(\widehat{\boldsymbol{\theta}}_n \pm \epsilon_n \Pi_n \boldsymbol{\iota}^* - \boldsymbol{\theta}_0; O) \Big\} \\
& + \mathcal{P} \Big\{ \rho(\widehat{\boldsymbol{\theta}}_n - \boldsymbol{\theta}_0; O) - \rho(\widehat{\boldsymbol{\theta}}_n \pm \epsilon_n \Pi_n \boldsymbol{\iota}^* - \boldsymbol{\theta}_0; O) \Big\} \\
= & \mp \epsilon_n \mathcal{P} \ell'(\boldsymbol{\theta}_0; O)[\boldsymbol{\iota}^*] \mp I_1 + I_2 + I_3.
\end{aligned}$$

Following the same arguments as presented on Page 9 of the supplementary materials in Zhou et al. (2017) [12], we obtain that

$$I_1 = \epsilon_n \times o_p(n^{-1/2})\,,$$

$$I_2 = (\mathbb{P}_n - \mathcal{P}) \Big\{ \rho(\widehat{\boldsymbol{\theta}}_n - \boldsymbol{\theta}_0; O) - \rho(\widehat{\boldsymbol{\theta}}_n \pm \epsilon_n \Pi_n \boldsymbol{\iota}^* - \boldsymbol{\theta}_0; O) \Big\} = \epsilon_n \times o_p(n^{-1/2})\,,$$

and

$$I_3 = \mathcal{P} \Big\{ \rho(\widehat{\boldsymbol{\theta}}_n - \boldsymbol{\theta}_0; O) - \rho(\widehat{\boldsymbol{\theta}}_n \pm \epsilon_n \Pi_n \boldsymbol{\iota}^* - \boldsymbol{\theta}_0; O) \Big\} = \pm \epsilon_n < \widehat{\boldsymbol{\theta}}_n - \boldsymbol{\theta}_0, \boldsymbol{\iota}^* > + \epsilon_n \times o_p(n^{-1/2})\,.$$

Together with the fact that $\mathcal{P}\ell'(\boldsymbol{\theta}_0; O)[\boldsymbol{\iota}^*] = 0$, we obtain

$$0 \leq \mathbb{P}_n\Big\{\ell(\widehat{\boldsymbol{\theta}}_n; O) - \ell(\widehat{\boldsymbol{\theta}}_n \pm \epsilon_n \Pi_n \boldsymbol{\iota}^*; O)\Big\} \pm \epsilon_n < \widehat{\boldsymbol{\theta}}_n - \boldsymbol{\theta}_0, \boldsymbol{\iota}^* > + \epsilon_n \times o_p(n^{-1/2})\,.$$

Hence it follows from the central limit theorem with $||\boldsymbol{\iota}^*||^2 = ||\ell'(\boldsymbol{\theta}_0; O)[\boldsymbol{\iota}^*]||^2$ that

$$\sqrt{n} < \widehat{\boldsymbol{\theta}}_n - \boldsymbol{\theta}_0, \boldsymbol{\iota}^* >= \sqrt{n}\,(\mathbb{P}_n - \mathcal{P})\{\ell'(\boldsymbol{\theta}_0; O)[\boldsymbol{\iota}^*]\} + o_p(1) \Rightarrow \mathcal{N}(\mathbf{0}, ||\boldsymbol{\iota}^*||^2)\,.$$

Based on this normality and the above discussions, the semiparametric efficiency result holds following the arguments in Theorem 4 of Shen (1997) [7].

Then, we need to check the conditions for the Riesz representation theory. Following the idea from section 3.2 of Chen et al. (2006) [2]. By the definition of $\ell(\boldsymbol{\theta}, O^{\psi})$, we have

$$\ell'(\boldsymbol{\theta}_0; O)[\boldsymbol{\iota}] = \lim_{s\to 0} \frac{\ell(\boldsymbol{\theta}_0 + s\boldsymbol{\iota}; O) - \ell(\boldsymbol{\theta}_0; O)}{s} = \frac{\partial \ell(\boldsymbol{\theta}_0; O)}{\partial \boldsymbol{\eta}}[\boldsymbol{\iota}_{\eta}] + \sum_{k=1}^{K} \frac{\partial \ell(\boldsymbol{\theta}_0; O)}{\partial \Lambda_k}[\boldsymbol{\iota}_{\Lambda_k}]\,.$$

Define $\{\widetilde{\Lambda}_k^*;\, k = 1, \ldots, K\}$ be the solution to

$$\inf_{\{\Lambda_k^*;\, k=1,\ldots,K\}} \mathbb{E}\Big\{\frac{\partial \ell(\boldsymbol{\theta}_0; O)}{\partial \boldsymbol{\eta}} \boldsymbol{e}_k - \sum_{k=1}^{K} \frac{\partial \ell(\boldsymbol{\theta}_0; O)}{\partial \Lambda_k}[\Lambda_k^*]\Big\}^2,$$

the k-th element of $l^*(\boldsymbol{\eta}_0)$ as

$$\frac{\partial \ell(\boldsymbol{\theta}_0; O)}{\partial \boldsymbol{\eta}} \boldsymbol{e}_k - \sum_{k=1}^{K} \frac{\partial \ell(\boldsymbol{\theta}_0; O)}{\partial \Lambda_k}[\widetilde{\Lambda}_k^*]\,,$$

and

$$I_*(\boldsymbol{\eta}_0) = \mathbb{E}\Big\{l^*(\boldsymbol{\eta}_0)^{\otimes 2}\Big\}\,.$$

Furthermore, by following similar calculations in Hu et al. (2017) [4], we can obtain that

$$||\boldsymbol{\iota}^*||^2 = \sup_{\boldsymbol{\iota}\in\Upsilon:||\boldsymbol{\iota}||>0} \frac{|h'(\boldsymbol{\theta}_0)[\boldsymbol{\iota}]|^2}{||\boldsymbol{\iota}||^2} = \sup_{\boldsymbol{\iota}\in\Upsilon:||\boldsymbol{\iota}||>0} \frac{|\boldsymbol{\alpha}^\top \boldsymbol{\iota}_{\eta}|^2}{||\boldsymbol{\iota}||^2} = \boldsymbol{\alpha}^\top \Big[\mathbb{E}\{l^*(\boldsymbol{\eta}_0)\}^{\otimes 2}\Big]^{-1} \boldsymbol{\alpha} = \boldsymbol{\alpha}^\top I_*^{-1}(\boldsymbol{\eta}_0)\boldsymbol{\alpha}.$$

Then, combine the above results, we have that $\sqrt{n}\{\widehat{\boldsymbol{\eta}}_n - \boldsymbol{\eta}_0\} \Rightarrow \mathcal{N}(\mathbf{0}, \boldsymbol{\Sigma})$ where $\boldsymbol{\Sigma} = I_*^{-1}(\boldsymbol{\eta}_0)$. This completes the proof of the asymptotic normality.

□

2 Additional Simulation Results

Following the reviewers' suggestion, we re-implemented the proposed sieve procedure using B-splines in place of Bernstein polynomials. Specifically, we replicated the setting of Table 1 in the main paper with $n = 400$ and employed a cubic B-spline basis with two choices for the number of knots (2 and 5). The results, reported in Table S1, indicate that the B-spline and Bernstein implementations perform comparably well. We also examined robustness to the observation process by varying the censoring

rate and the inter-visit gap. Again replicating the Table 1 setting with $n = 400$ and $\zeta_0 = 0.20$, we modified the visit schedule to right censoring rates of 15% and 30%, and average inter-visit gaps of 0.25 and 0.50. The corresponding results, presented in Table S2, again demonstrate strong performance of the proposed method across these alternative designs.

Table S1: Comparison results for the proposed method using B-splines versus Bernstein polynomials.

		Bernstein		Cubic B-spline (2)		Cubic B-spline (5)	
MR	Para	Bias	SD	Bias	SD	Bias	SD
40%	$\beta_{1,1}$	-0.0128	0.0946	-0.0137	0.0946	-0.0197	0.0929
	$\beta_{1,2}$	0.0158	0.1869	0.0152	0.1883	0.0188	0.1930
	$\beta_{2,1}$	0.0226	0.1115	0.0208	0.1128	0.0262	0.1143
	$\beta_{1,2}$	-0.0123	0.2278	-0.0089	0.2293	-0.0251	0.2242
	γ_0	-0.0131	0.2327	-0.0157	0.2325	-0.0004	0.2344
	γ_1	0.0066	0.1408	0.0083	0.1412	0.0120	0.1417
	γ_2	0.0084	0.3002	0.0074	0.3002	-0.0088	0.2927
60%	$\beta_{1,1}$	-0.0126	0.0871	-0.0136	0.0878	-0.0199	0.0842
	$\beta_{1,2}$	0.0137	0.1779	0.0143	0.1807	0.0124	0.1822
	$\beta_{2,1}$	0.0205	0.0998	0.0189	0.1005	0.0200	0.0987
	$\beta_{1,2}$	-0.0149	0.1995	-0.0135	0.2003	-0.0130	0.2048
	γ_0	-0.0017	0.2005	-0.0030	0.2005	-0.0078	0.2006
	γ_1	0.0074	0.1222	0.0082	0.1222	0.0103	0.1222
	γ_2	0.0009	0.2601	0.0005	0.2600	0.0111	0.2582

Table S2: Simulation results based on the proposed method under different settings.

Inter-visit		Censoring Rate = 15%				Censoring Rate = 30%			
Gap	Para	Bias	SD	ESE	CP	Bias	SD	ESE	CP
0.25	$\beta_{1,1}$	-0.0189	0.1069	0.1037	0.949	-0.0153	0.1603	0.1426	0.913
	$\beta_{1,2}$	0.0232	0.2107	0.2030	0.944	0.0136	0.2879	0.2649	0.944
	$\beta_{2,1}$	0.0115	0.1166	0.1076	0.933	0.0101	0.1483	0.1309	0.927
	$\beta_{1,2}$	-0.0164	0.2359	0.2235	0.940	-0.0155	0.3040	0.2774	0.931
	γ_0	-0.0186	0.2331	0.2353	0.964	-0.0226	0.2859	0.2717	0.933
	γ_1	0.0069	0.1447	0.1370	0.944	0.0038	0.1600	0.1550	0.940
	γ_2	0.0016	0.3007	0.3017	0.948	0.0265	0.3489	0.3296	0.943
0.50	$\beta_{1,1}$	-0.0160	0.1186	0.1107	0.936	-0.0047	0.1527	0.1372	0.912
	$\beta_{1,2}$	0.0194	0.2278	0.2142	0.939	0.0205	0.2890	0.2573	0.936
	$\beta_{2,1}$	0.0250	0.1308	0.1221	0.949	0.0068	0.1495	0.1321	0.931
	$\beta_{1,2}$	-0.0323	0.2617	0.2492	0.942	-0.0281	0.3159	0.2801	0.925
	γ_0	-0.0177	0.2356	0.2418	0.954	-0.0178	0.2890	0.2666	0.937
	γ_1	0.0095	0.1457	0.1407	0.947	0.0030	0.1667	0.1521	0.933
	γ_2	0.0119	0.3000	0.3096	0.961	0.0381	0.3423	0.3279	0.934

References

[1] Arcones, M. A. and Giné, E. (1993). Limit theorems for u-processes. *The Annals of Probability*, 21(3):1494–1542.

[2] Chen, X., Fan, Y., and Tsyrennikov, V. (2006). Efficient estimation of semiparametric multivariate copula models. *Journal of the American Statistical Association*, 101(475):1228–1240.

[3] Du, M., Lou, Y., and Sun, J. (2024). Estimation and variable selection for interval-censored failure time data with random change point and application to breast cancer study. *Journal of the American Statistical Association*, (just-accepted):1–23.

[4] Hu, T., Zhou, Q., and Sun, J. (2017). Regression analysis of bivariate current status data under the proportional hazards model. *Canadian Journal of Statistics*, 45(4):410–424.

[5] Lorentz, G. G. (1986). *Bernstein Polynomials.* New York: Chelsea Publishing Co.

[6] Lou, Y., Ma, Y., Sun, J., Wang, P., and Ye, Z. (2025). Instrumental variable estimation of complier casual treatment effects with interval-censored competing risks data. *Biometrics*, 81(1):ujaf010.

[7] Shen, X. (1997). On methods of sieves and penalization. *The Annals of Statistics*, 25(6):2555–2591.

[8] Shen, X. and Wong, W. H. (1994). Convergence rate of sieve estimates. *The Annals of Statistics*, 24(2):580–615.

[9] van de Geer, S. (2000). *Empirical Processes in M-estimation*, volume 6. Cambridge University Press.

[10] van der Vaart, A. W. (2000). *Asymptotic statistics*, volume 3. Cambridge University Press.

[11] van der Vaart, A. W. and Wellner, J. (1996). *Weak Convergence and Empirical Processes: with Applications to Statistics.* Springer Science & Business Media.

[12] Zhou, Q., Hu, T., and Sun, J. (2017). A sieve semiparametric maximum likelihood approach for regression analysis of bivariate interval-censored failure time data. *Journal of the American Statistical Association*, 112(518):664–672.
